# Effectiveness of auriculotherapy and laser acupuncture in *hyperemesis gravidarum*: a randomized clinical trial*

**DOI:** 10.1590/1518-8345.7235.4771

**Published:** 2026-01-19

**Authors:** Lais de Lima Oliva, Renne Rodrigues, Keli Regiane Tomeleri da Fonseca Pinto, Sonia Silva Marcon, Catia Campaner Ferrari Bernardy

**Affiliations:** 1 Universidade Estadual de Londrina, Londrina, PR, Brazil. Universidade Estadual de Londrina PR Londrina Brazil; 2 Universidade Federal da Fronteira do Sul, Curso de Medicina, Chapecó, SC, Brazil. Universidade Federal da Fronteira do Sul Curso de Medicina SC Chapecó Brazil; 3 Universidade Estadual de Londrina, Departamento de Saúde Coletiva, Londrina, PR, Brazil. Universidade Estadual de Londrina Departamento de Saúde Coletiva PR Londrina Brazil; 4 Universidade Estadual de Londrina, Departamento de Enfermagem, Londrina, PR, Brazil. Universidade Estadual de Londrina Departamento de Enfermagem PR Londrina Brazil; 5 Universidade Estadual de Maringá, Departamento de Enfermagem, Maringá, PR, Brazil. Universidade Estadual de Maringá Departamento de Enfermagem PR Maringá Brazil

**Keywords:** Complementary Therapies, Low-Level Light Therapy, Auriculotherapy, Pregnancy, Morning Sickness, Quality of Life

## Abstract

to evaluate the effectiveness of auriculotherapy and laser auriculotherapy on nausea, vomiting, and quality of life in pregnant women.

a randomized, parallel, factorial, double-blind study was conducted with 100 pregnant women, allocated into four groups (25 in each): intervention (G1- auriculotherapy and G2- laser auriculotherapy), control (G3), and placebo with cotton (G4), all followed for seven days for outcome analysis. The Pregnancy Unique Quantification of Emesis Score and the Degree of Auricular Palpation were used to analyze nausea and vomiting, and the Health-Related Quality of Life Questionnaire for Nausea and Vomiting of Pregnancy was used to assess quality of life.

compared to the placebo group, participants in G1 and G2 reduced (p<0.050) their Pregnancy Unique Quantification of Emesis scores by -1.14 (95% CI: -2.11; -0.17) and -1.3 (95% CI: -2.24; -0.41) and the Health-Related Quality of Life Questionnaire for Nausea and Vomiting of Pregnancy by -34.81 (95% CI: -62.98; -0.95) and -31.97 (95% CI: -62.98; -0.95), respectively. The use of antiemetics was higher in the control and placebo groups.

both types of intervention demonstrated a significant reduction in nausea/vomiting and the use of allopathic medications, an improvement in quality of life, and potential acceptance of laser auriculotherapy by pregnant women. Brazilian Registry of Clinical Trials RBR-4wtq84v.

## Highlights

**(1)** The use of laser auriculotherapy is as effective as auriculotherapy for NVP. **(2)** Laser acupuncture and auriculotherapy reduce the use of allopathic medications for NVP. **(3)** Laser auriculotherapy is a painless option for treating NVP.

## Introduction

Nausea and vomiting are common in early pregnancy and can have negative effects on pregnancy, depending on their intensity and frequency, as well as interfering with women’s ability to work and compromising their quality of life during this period^(1)^. They are present in 85% of cases, but are most frequent between the 5th and 9th weeks of pregnancy (90%), gradually decreasing until they become infrequent after the 20th week. Cases requiring drug treatment are close to 10%, and approximately 1.1% of pregnancies progress to the severe form called hyperemesis gravidarum^(2)^.

Given the high incidence of Nausea and Vomiting of Pregnancy (NVP), the recurring daily limitations of this clinical condition, and the possible negative implications that the use of medication during pregnancy can have for women, ranging from concerns about the health and development of the fetus^(3)^ to economic issues or even side effects on the physical and/or mental health of affected women, it is essential to seek alternatives that enable a non-pharmacological approach, even if it has a positive effect on reducing symptoms and, consequently, on the quality of life of these women. It is also important that the proposed alternative be low-cost, easily accepted by users, and feasible for use in public health services, which are responsible for the care and monitoring of the vast majority of women during pregnancy in Brazil.

Acupuncture is a non-pharmacological approach that can help treat these complaints and is even recommended by the Brazilian Federation of Gynecology and Obstetrics Associations (FEBRASGO)^(2)^. It is a complementary therapy characterized by the stimulation of specific points using needles or lasers. When applied to the ear, it is called auriculotherapy^(2)^ or laser auriculotherapy, used in people who are sensitive to needle discomfort or have coagulation disorders.

The auricular points are connected to other structures in the body by sensory endings of the vagus nerve. Vagal neurons project one end to the organs, and the other connects to the nucleus of the solitary tract in the brainstem, which originates the cranial nerves and transmits action potentials to control organ function. After auricular stimulation, the production of neurotransmitters that modulate the brain-gut axis is triggered, reducing visceral hypersensitivity and regulating gastric motility^(4-6)^, consequently reducing the manifestation of symptoms such as nausea and vomiting.

Acupuncture is included in the National Policy on Integrative and Complementary Practices (PNPIC, as per its acronym in Portuguese)^(7)^ as one of the practices that can be offered to the population at all levels of care within the Unified Health System. However, it is in the context of primary care that it has been effectively incorporated, with nurses responsible for a significant number of procedures^(8)^.

The benefits of acupuncture have often been reported in the literature for different problems and audiences. Considering women during pregnancy, the national literature has reported the use of acupuncture in cases of nausea and vomiting^(9)^ and auriculotherapy for relief of low back pain^(10-12)^, stress^(13)^, anxiety^(14)^, and insomnia^(15)^. Internationally, a review study that included 13 Randomized Clinical Trials (RCTs) considered the effectiveness of auriculotherapy for nausea and vomiting during pregnancy to be inconclusive, either due to the size of the samples or the methodological aspects adopted in the studies^(16)^. Another RCT involving 128 pregnant women concluded that auriculotherapy significantly reduced nausea but not vomiting, a result that the authors attributed to the fact that participants had to press the points three times a day for at least 30 seconds for four days^(17)^.

It should be noted that no RCTs evaluating the effect of laser auriculotherapy on the control of NVP were found in the available literature. The lack of robust scientific evidence on the effectiveness of auriculotherapy and laser auriculotherapy among pregnant women with nausea and vomiting highlights the novelty of this study, whose results and protocol may contribute to the professional practice of nurses. It is worth remembering that the use of Alternative and Complementary Practices (ACPs), which include acupuncture, is recognized by the Federal Nursing Council through Resolution No. 739 of 2024^(18)^. In view of the above, the objective was defined as evaluating the effectiveness of auriculotherapy and laser auriculotherapy on nausea, vomiting, and quality of life in pregnant women.

## Method

### Study type

This is a randomized, parallel, factorial, double-blind clinical trial with a 1:1 allocation ratio^(19)^.

### Study setting

The study was conducted in three Basic Health Units (BHUs) in a municipality in northern Paraná, selected because they had the highest flow of pregnant women and offered shared prenatal consultations between physicians and obstetric nursing residents. Each BHU saw an average of 30 pregnant women per month, and the three had similar territorial coverage.

### Population and selection criteria

The participants were pregnant women at usual obstetric risk who were receiving prenatal care at the three selected primary health care units. Eligible participants were women over 18 years of age, with a gestational age between 5 and 16 weeks, regardless of parity, who presented mild or moderate symptoms of nausea and/or vomiting according to the Pregnancy-Unique Quantification of Emesis (PUQE) score (Mild < 6; Moderate between 7 and 11). Exclusion criteria included pregnant women with a diagnosed intellectual disability and suicidal ideation, coagulation disorders, pharmacological treatment for psychosomatic conditions such as anxiety and depression, hyperthyroidism, dermatological lesions on the auricle, unexplained fever, multiple gestation, gestational trophoblastic disease, hyperemesis gravidarum, antiemetic use, diarrhea, pregnancies with fetuses diagnosed with Down syndrome, and women with fear of receiving the semipermanent needle.

### Sample definition

The sample calculation was performed using StataCorp, College Station (Stata), version 16.0, considering the following parameters: 4 intervention groups: Power: 0.80 (80%), alpha: 0.05, moderate effect size of differences (ƞ2): 0.15, and intragroup error variance: 1.0. Considering the four intervention groups, an effect size measure appropriate for ANOVA models (ƞ2)^(20)^ was chosen. The parameters used considered the recommendations for systematic sampling that would allow the analyses performed to be sufficient to detect clinically relevant differences, avoiding type I and II errors^(21)^. Based on these parameters, a minimum sample of 80 participants (n=20 per group) was determined, and 25% was added for possible losses, totaling 100 participants (25 in each group). The sampling plan followed the guidelines of the Consolidated Standards of Reporting Trials (CONSORT).

Simple randomization into four groups was performed by an assistant researcher using the website https://www.randomizer.org, and the results were placed in opaque envelopes that were only opened at the time of treatment and in the presence of the acupuncturist researcher and the patient.

### Instruments

The PUQE is a self-report test used to assess the severity of NVP, translated and validated by FEBRASGO, consisting of three five-point Likert scale questions (Figure 1), with three cut-off times (last 6, 12, or 24 hours). The longest time (24 hours) is the most commonly used, with less than 6 hours used to assess the therapeutic effect of an intervention and to diagnose the intensity of NVP.^(2)^.

**Figure 1-:** Determination of the severity of nausea and vomiting of pregnancy. Pregnancy Unique Quantification of Emesis score. Paraná, Brazil, 2023

1 – How long have you felt nauseous in the last 24 hours?
Never (1) – Up to 4 hours (2) – Up to 8 hours (3) – Up to 12 hours (4) – More than 12 hours (5)
2 – How many vomiting episodes have you had in the last 24 hours?
None (1) – One episode (2) – Up to 3 episodes (3) – Up to 4 episodes (4) – More than five (5)
3 – How many times have you experienced intense salivation and retching in the last 24 hours?
None (1) – Up to 3 times (2) – Up to 5 times (3) – Up to 8 times (4) – All the time (5)
**Classification** – Score ≤ 6, mild form; between 7 and 11, moderate form; ≥ 12, severe form

Source: Brazilian Federation of Gynecology and Obstetrics Associations^(2)^

The Health-Related Quality of Life Questionnaire for Nausea and Vomiting of Pregnancy (NVPQOL) is a questionnaire that assesses the quality of life of pregnant women with NVP. It consists of 30 questions distributed across four domains: physical symptoms and their aggravating factors, exhaustion, emotional factors, and limitations. Responses are presented on a 7-point Likert scale, where 1 represents the absence of the symptom in the last seven days and 7 represents the presence of the symptom all the time. The total score can range from 30 to 210, with the lower the score, the higher the quality of life^(22-23)^. The version used was validated and translated into Brazilian Portuguese in 2021 and showed strong internal consistency (Cronbach’s α: 0.95), strong intra-rater reliability, and test-retest reliability (P < 0.0; intraclass correlation coefficient: 0.89; confidence interval: 0.791-0.945)^(23)^.

Reactivity to auricular palpation was also used, classified into three levels: Grade I – pain reported verbally; Grade II – pain expressed by facial expression, blinking, or frowning; Grade III – patient attempts to prevent the examination by turning their head away or placing their hand on the examiner’s hand. This classification assesses the patient’s progress in terms of sensitivity. It is expected that, as treatment progresses, the patient will become less sensitive to the stimuli of the examination^(24)^. For auricular palpation, a probe was used to identify the pressure pain threshold. This instrument has a smooth, spherical tip with a diameter of two millimeters.

For laser auriculotherapy, the Therapy EC device was used, manufactured in 2018 by the enterprise Dental Manufacturing Company (DMC), which has two laser diode emitters, one red and the other infrared, and an individual fiber optic delivery system for each emitter. The useful diameter of the optical fibers is 600 µm per fiber. It also has a low-intensity aiming beam to indicate the anticipated point of impact of the infrared laser. The useful power of the emitter is 100 mW ± 20%, with a continuous wave and pulse duration of 10 seconds per Joule (J). Protective glasses were used during laser application by pregnant women and the researcher.

The irradiation parameters used were: red laser with a wavelength of 660 nm ± 10 nm, energy of 3 J per auricular point, which is equivalent to an energy density of 107 J/cm^2^ per point. Thus, seven auricular points were irradiated in a single session, totaling accumulated energy of 21 J in the right auricle. It took 3.5 minutes to apply it to the 7 points. The area of the auricular point corresponds to approximately 0.028 cm²^(25-26)^.

The auriculotherapy group (G1) received the application of a semi-permanent, stainless steel, sterilized, disposable needle, manufactured by *Complementar Agulhas*, ANVISA Registration No. 80731150002, measuring 0.20 x 1.5 mm, which was attached to a beige micropore tape measuring 0.8 x 0.8 cm. Participants in the placebo group (G4) received a cotton ball measuring less than 1.7 mm attached with micropore tape.

### Study period and data collection

Data collection took place between April and October 2022, in a room exclusively reserved for research consultations at the units themselves.

After sample calculation, obstetric nursing residents working in the BHUs referred a total of 117 pregnant women to the acupuncturist so that the statistically estimated population could be reached. Of these, 17 were not included in the study, 13 due to unavailability due to work and four due to difficulty in traveling. The 100 participants were allocated equally and randomly into four groups (N=25 per group): intervention (G1, semi-permanent needle and G2, laser auriculotherapy), G3 control, and G4 placebo. The women were followed up for seven days to evaluate the outcome.

The selection of pregnant women, according to the established criteria, was carried out by the residents, who explained the nature of the research and scheduled an appointment with the acupuncturist (an obstetric nurse duly trained to apply the two intervention techniques).

On the day of the consultation with the acupuncturist, before the appointment, the residents conducted a preliminary interview, during which they also requested the signing of a Free and Informed Consent Form (FICF). During the interview, a two-part instrument was used: the first part consisted of questions about sociodemographic and obstetric characteristics, and the second part consisted of two questionnaires, the PUQE^(2)^ and the NVPQOL^(22-23)^. These instruments were administered by the residents at the first appointment and by the BHU nurse at the second appointment (seven days after the first visit), before the acupuncturist’s appointment. The completed forms were sent for analysis by the statistician. Thus, residents and statisticians were blinded in the study.

All participants had their right auricle evaluated by the acupuncturist, regardless of the group to which they belonged. The inspection could show the presence of spots, hypervascularization, scaling, nodules, or papules. After the visual inspection, auricular palpation was performed with the palpator.

The protocol used for nausea and vomiting (NV) was made up of the following points: Stomach, Cardia, Central Nervous System, Autonomic Nervous System, Kidney, Subcortex, and Occipital, determined based on previous studies and basic literature on auriculotherapy^(14,24,27-28)^. The acupuncturist palpated the acupoints to identify pressure sensitivity on the ear surface and classified the reactivity to palpation at two points in time: at the first appointment (Palpation 1) and at the follow-up appointment seven days later (Palpation 2).

Semi-permanent needles were applied to the right auricle of the G1 participants, at the NV points determined in the protocol. A single session was performed, with a follow-up visit on the seventh day. The pregnant women were instructed not to stimulate the points, to keep the auricle dry, to inspect the integrity of the micropore tape securing the needle, and to observe reactions such as local redness, pain, dizziness, tachycardia, or sweating, and to contact the acupuncturist immediately if any of these occurred. In this contact, they would be instructed to return for reinspection, adjustment, or removal of the semi-permanent needle, in order to minimize exclusion bias. However, no participant needed to contact the acupuncturist.

The G2 participants received the same punctual application as those in G1 and returned on the seventh day for reassessment. This group had no material attached to the auricular pavilion and did not receive any stimulation between visits. The participants in G3 were seen individually by the acupuncturist, who performed an auricular inspection and palpation and gave them instructions on how to fill out the PUQE score and return on the seventh day for reassessment.

Participants in G4 received a single application of a cotton ball to the right auricle at seven random points determined by anatomical rationale (knee, ankle, elbow, leg, arm, neck, and lung)^(24,27)^, returning on the seventh day for reassessment. They were instructed not to apply pressure or manipulate the site, to inspect the integrity of the micropore and check for allergic reactions, such as local redness. If they experienced this side effect, they were instructed to contact the acupuncturist and return for reinspection, adjustment, or removal of the micropore with the cotton ball. The members of this group also did not need to contact the acupuncturist.

The acupuncturist and the participants in the four groups were not blinded due to the physical differences between the interventions. The participants in the four groups, in addition to responding to the PUQE Score at the first visit (PUQE 1), received two copies of the instrument and were instructed to complete them at the 24th (PUQE 2) and 96th (PUQE 3) hours after the first visit to the acupuncturist.

At the second appointment, seven days later, to classify the degree of auricular sensitivity, the acupuncturist pressed the same points used for NV, and the PUQE Score (4) and NVPQOL (2) were applied again by the BHU nurse.

The assessment of adverse effects was performed in a non-systematic manner, and possible unfavorable outcomes were monitored through questions directed at the presence of possible signs and symptoms when receiving the interventions.

All participants were instructed that they could take 10 mg of metoclopramide orally every 8 hours if they could not tolerate the symptoms of nausea and/or vomiting^(2)^. A pack of medication was given to participants at their first appointment, and the remaining pills in the pack were counted on their return on the seventh day.

To avoid attrition bias, the acupuncturist contacted participants daily by phone or messaging app (WhatsApp), including G3 participants, during the study period to ensure that the protocol was followed.

Although some women in G1 reported discomfort due to the presence of the needles, there were no dropouts, as all remained with the needles until the seventh day. There were no adverse effects on the micropore tape that secured the needles and cotton balls, as all remained intact during the week of intervention, and participants reported no symptoms of skin irritation.

### Data analysis

Descriptive data analysis was performed using absolute numbers (n) and relative percentages (%) for categorical variables, as well as means and minimum and maximum values or 95% confidence intervals (95% CI) for continuous variables. Considering the non-normal distribution of data, based on the Shapiro-Wilk test, the Kruskal-Wallis test (Post-Test: Dunn) was used to compare the groups in relation to continuous data. The Chi-Square test was used for categorical data.

Additionally, the analysis of the association between the intervention groups and the PUQE and NVPQOL scale scores was performed using generalized estimating equation (GEE) models for related repeated measures, with estimation of the beta coefficient (β) and 95% CI. The following parameters were used: normal distribution of the dependent variable, linear, independent matrix, and robust variance. Models were generated using both the control group and the placebo as references. Finally, models were adjusted for possible confounding factors, such as age, gestational age, body mass index, parity, and number of consultations. Statistical analysis was performed using the Statistical Package for the Social Sciences software, adopting a 95% confidence interval and a 5% significance level (p<0.05) for all tests applied.

### Ethical aspects

This RCT was approved by the Human Research Ethics Committee of the State University of Londrina under opinion number 5.091.470 and approved on the virtual platform of the Brazilian Registry of Clinical Trials under code RBR-4wtq84v, following the CONSORT recommendations. Participants were informed about the research and the confidentiality of the data collected and began participating after signing the informed consent form. The study was funded by the main researcher and the advisor’s own resources.

## Results

One hundred pregnant women were evaluated for the benefits and harms of the therapies, with no loss to follow-up or responses that could compromise the representativeness of the sample or the results of the statistical analyses.

The mean age of the pregnant women was 26.9 years (±6.4), with a minimum of 18 and a maximum of 40 years. their average body mass index (BMI) was 25.1 (±3.4), 81% of them had a partner, 68% had completed high school, with an average family income of 3 minimum wages, and most (76%) were engaged in paid work. Regarding the obstetric data of the current pregnancy, 60% were multiparous, with a mean gestational age of 8 weeks, and 99% had already registered for prenatal care and had at least one consultation. There was no statistical difference between the groups in terms of sociodemographic and obstetric variables (Table 1).

**Table 1-:** Distribution of socioeconomic, demographic, and obstetric data of pregnant women with nausea and vomiting (n = 100). Paraná, Brazil, 2023

**Variables**	**Auriculotherapy (G1)**	**Laser (G2)**	**Control (G3)**	**Placebo (G4)**	**p-value**
Age (full years)*	27.1 (18; 40)	26.2 (18; 40)	28.1 (18; 38)	26.2 (18; 40)	0.688
BMI (in Kg/m²)*	24.8 (20; 34)	25.2 (21; 32)	25.6 (20; 37)	24.9 (20; 34)	0.587
Gestational age (weeks)*	8.4 (6; 13)	8.3 (5; 12)	7.9 (5; 13)	9.1 (5; 12)	0.319
No. of people in the household*	3.2 (1; 5)	2.9 (2; 4)	2.9 (2; 6)	3.0 (2; 5)	0.378
Education ^†^					0.794
Incomplete high school or lower	3 (18.8)	3 (18.8)	4 (25.0)	6 (37.4)	
Complete high school	17 (25.0)	18 (26.5)	16 (23.5)	17 (25.0)	
Complete higher education	5 (31.3)	4 (25.0)	5 (31.3)	2 (12.4)	
Marital status ^†^					0.181
With a partner	17 (21.0)	20 (24.7)	23 (28.4)	21 (25.9)	
Without a partner	8 (42.1)	5 (26.3)	2 (10.5)	4 (21.1)	
Family income (minimum wages) ^‡^					0.643
1	1 (11.1)	1 (11.1)	3 (33.3)	4 (44.5)	
2	2 (11.1)	5 (27.8)	5 (27.8)	6 (33.3)	
3	11 (27.5)	11 (27.5)	8 (20.0)	10 (25.0)	
4	7 (31.8)	4 (18.2)	7 (31.8)	4 (18.2)	
5 to 6	4 (36.4)	4 (36.4)	2 (18.2)	1 (9.2)	
Paid activity ^†^					0.625
Yes	21 (27.6)	19 (25.0)	17 (22.4)	19 (25.0)	
No	4 (16.7)	6 (25.0)	8 (33.3)	6 (25.0)	
Parity ^†^					0.506
Primiparous	10 (25.0)	11 (27.5)	7 (17.5)	12 (30.0)	
Multiparous	15 (25.0)	14 (23.3)	18 (30.0)	13 (21.7)	
Number of appointments ^†^					0.640
0	0 (-)	1 (100.0)	0 (-)	0 (-)	
1	23 (26.1)	20 (22.7)	23 (26.1)	22 (25.1)	
2	2 (18.2)	4 (36.4)	2 (18.2)	3 (27.2)	

*Mean (minimum value; maximum value); ^†^n (%); ^‡^Current minimum wage = R$ 1,212.00, Brazil, 2022

The PUQE and NVPQOL scores before the interventions ranged from 8.2 to 8.7 and from 121.5 to 137.0, respectively, with no statistical difference between the groups. After the interventions, there was a statistically significant reduction in PUQE 4 and NVPQOL 2 for the auriculotherapy and laser auriculotherapy groups, indicating a significant reduction in symptoms and improvement in quality of life 4 days after the interventions (Table 2).

Before the intervention, most of the pregnant women in the four groups had Grade II palpation. Pregnant women who received auriculotherapy and laser auriculotherapy showed a statistically significant reduction in the degree of palpation, moving to Grade I at consultation 2, which means stabilization of those points and balance of the corresponding organ. There were no reports of adverse effects in the intervention, placebo, and control groups.

**Table 2-:** Assessment of PUQE* and NVPQOL^†^ scores, degree of sensitivity to auricular palpation, and medication use among pregnant women with nausea and vomiting (n = 100). Paraná, Brazil, 2023

**Variables**	**Auriculotherapy (G1)**	**Laser (G2)**	**Control (G3)**	**Placebo (G4)**	**p-value**
PUQE* 1 ^‡^	8.4 (7.8; 9.1)	8.2 (7.4; 8.9)	8.7 (8.0; 9.3)	8.4 (7.8; 9.1)	0.782
PUQE* 2 ^‡^	7.8 (7.1; 8.5)	7.5 (6.7; 8.2)	8.7 (8.1; 9.4)	8.5 (7.8; 9.2)	0.042
PUQE* 3 ^‡^	6.9 (6.3; 7.5)	6.8 (6.1; 7.5)	8.5 (7.9; 9.1)	8.4 (7.8. 9.1)	<0.001
PUQE* 4 ^‡^	5.9 (5.3; 6.5)	5.8 (5.1; 6.5)	8.5 (7.9; 9.1)	8.6 (7.9; 9.2)	<0.001
NVPQOL ^†^ 1 ^‡^	121.5	127.8	134.6	137.0	0.075
	(114.0; 128.9)	(117.5; 138.2)	(126.4; 142.8)	(127.9; 146.2)	
NVPQOL ^†^ 2 ^‡^	86.0	85.8	133.4	139.6	<0.001
	(79.4; 92.7)	(77.8; 93.8)	(124.5; 142.4)	(131.1; 148.1)	
Palpation 1 ^§^					0.387
Grade I	0 (-)	0 (-)	0 (-)	1 (100.0)	
Grade II	25 (25.3)	25 (25.3)	25 (25.3)	24 (24.1)	
Palpation 2 ^§^					<0.001
Grade I	14 (43.8)	16 (50.0)	1 (3.1)	1 (3.1)	
Grade II	11 (16.2)	9 (13.2)	24 (35.3)	24 (35.3)	
Metoclopramide dosage used (mg) ^*^	2.8	5.2	40.0	42.4	<0.001
	(0.6; 5.0)	(1.6; 8.8)	(28.2; 51.8)	(30.2; 54.6)	
Continued use of ferrous sulfate ^§^					0.243
Yes	20 (24.1)	20 (24.1)	19 (22.9)	24 (28.9)	
No	5 (29.4)	5 (29.4)	6 (35.3)	1 (5.9)	

*PUQE = Pregnancy Unique Quantification of Emesis; ^†^NVPQOL = Health-Related Quality of Life Questionnaire for Nausea and Vomiting of Pregnancy; ^‡^Mean (95% confidence interval); ^§^n (%)

It was observed that pregnant women in G3 and G4 made statistically greater use of metoclopramide than the intervention groups (#tbl:3 Table 2). In addition, most participants made continuous use of ferrous sulfate at a dosage of 40 mg/day to prevent gestational anemia, with no statistical difference between the groups.

In the analysis of the overall NVPQOL score before the interventions, there were higher scores in items 1, 4, 8, 15, and 29, which are, respectively, nausea, urge to vomit, feeling worse with certain smells, feeling less interest in sex, and difficulty cooking, indicating that NVP symptoms can affect various areas of pregnant women’s life and that of their families, and that smell can influence the worsening of symptoms. Analyzing the items that had the lowest scores before the intervention (6, 19, 20, and 21), which are, respectively, worsening of symptoms in the afternoon, feeling that your symptoms are not normal for pregnancy, feeling that you cannot enjoy pregnancy and that everything is an effort, they indicate that, despite the unpleasant symptoms, most pregnant women face NV as a physiological event of pregnancy.

Regarding the reduction in scores after the application of auriculotherapy and laser auriculotherapy, the items that showed the greatest reduction were 1, 4, 5, 8, and 9, which are, respectively, nausea, urge to vomit, loss of appetite, and feeling worse with certain smells or foods. These reduced scores indicate that the therapies were effective in reducing the symptoms. Two items showed little reduction with the interventions: item 6, which refers to worsening symptoms in the afternoon, which had already been scored low before the intervention, and item 14, about feeling emotional, which can be justified by the hormonal changes that occur in early pregnancy.

Participants in G1 and G2 showed statistically significant reductions in PUQE and NVPQOL scores in the raw models when compared to those in G3 and G4 (Table 3). The results were mostly replicated in a model adjusted for confounding factors, with the exception of G2 for NVPQOL, which lost statistical significance when compared to G3.

Compared to G4 in the adjusted model, groups G1 and G2 reduced (p<0.050) PUQE scores by -1.14, with a 95% confidence interval (95% CI: -2.11; -0.17) and -1.3 (95% CI: -2.24; -0.41), and NVPQOL scores by -34.81 (95% CI: -62.98; -0.95) and -31.97 (95% CI: -62.98; -0.95), respectively (Table 3).

**Table 3-:** Evaluation of the effectiveness of auriculotherapy and laser auriculotherapy in relation to PUQE* and NVPQOL^†^ scores among pregnant women with nausea and vomiting (n = 100). Paraná, Brazil, 2023

**Raw model**	**Control (G3)**	**Compared to the control group**		
		**Placebo** ^‡^ **(G4)**	**Auriculotherapy** ^‡^ **(G1)**	**Laser therapy** ^‡^ **(G2)**
PUQE*	0.00	-0.13 (-0.25; -0.01. 0.027)	-1.34 (-2.19; -0.49. 0.002)	-1.55 (-2.36; -0.74. <0.001)
NVPQOL ^†^	0.00	4.26 (1.68; 6.84. 0.001)	-30.28 (-51.01; -6.55. 0.012)	-27.22 (-55.52; 1.08. 0.059)
**Adjusted model** ^§^	**Control (G3)**	**Placebo** ^‡^ **(G4)**	**Auriculotherapy** ^‡^ **(G1)**	**Laser therapy** ^‡^ **(G2)**
PUQE*	0.00	-0.26 (-0.39; -0.13. <0.001)	-1.40 (-2.25; -0.54. 0.001)	-1.59 (-2.38; -0.79. <0.001)
NVPQOL ^†^	0.00	5.99 (3.26;8.73)	-28.82 (-52.64; -4.99. 0.018)	-25.97 (-54.25; 2.31. 0.072)
**Raw model**	**Placebo (G4)**	**Compared to the placebo group**		
		**Control** ^‡^ **(G3)**	**Auriculotherapy** ^‡^ **(G1)**	**Laser therapy** ^‡^ **(G2)**
PUQE*	0.00	0.13 (0.01; 0.25. 0.027)	-1.21 (-2.18; -0.24. 0.014)	-1.42 (-2.34; -0.50. 0.002)
NVPQOL ^†^	0.00	-4.26 (-6.84; -1.68)	-34.54 (-60.84; -8.24. 0.010)	-31.48 (-62.36; -0.60. 0.046)
**Adjusted model** ^§^	**Placebo (G4)**	**Control** ^‡^ **(G3)**	**Auriculotherapy** ^‡^ **(G1)**	**Laser therapy** ^‡^ **(G2)**
PUQE*	0.00	0.26 (0.13; 0.39. <0.001)	-1.14 (-2.11; -0.17. 0.022)	-1.32 (-2.24; -0.41. 0.005)
NVPQOL ^†^	0.00	-5.99 (-8.73; -3.26. <0.001)	-34.81 (-62.98; -0.95. 0.043)	-31.97 (-62.98; -0.95. 0.043)

*PUQE = Pregnancy Unique Quantification of Emesis; ^†^NVPQOL = Health-Related Quality of Life Questionnaire for Nausea and Vomiting of Pregnancy; ^‡^Beta (95% confidence interval. p-value); ^§^Model adjusted for age, gestational age, body mass index, parity, and number of consultations

## Discussion

Auriculotherapy and laser auriculotherapy were effective in reducing NVP and improving the quality of life of pregnant women. However, in the adjusted model, G2 compared to G3 did not show a statistically significant improvement in quality of life, although there was a reduction in NVPQOL scores, which leads us to suggest further research with a larger population.

The quality of life of pregnant women can be affected by their relationship with their partner and family. Marital conflicts can be considered etiological factors for anxiety and depression during this period, emotions that can exacerbate NVP symptoms^(29)^. The well-being of pregnant women may also be related to their active sex life during this period, but symptoms such as NV can negatively influence libido^(30)^, which, in the present study, was reported as less interest in sex.

Other factors may intensify the presence of NVP, such as hormonal issues, a diet high in fat or spicy foods, and certain smells, as the sense of smell is more acute in pregnant women in the first trimester. Given this, a healthy diet is associated with a lower risk of developing hyperemesis gravidarum^(31)^.

The heightened sense of smell in women during the first trimester of pregnancy is related to the cognitive processing of olfactory information and is reported to be more altered than taste. Thus, smell may have greater potential to trigger NVP symptoms^(31)^, which confirms the findings of the present study, as women reported that smelling certain odors causes more NV than eating certain foods.

One of the items with the lowest score before the intervention, and which showed virtually no change after the therapies, was the worsening of NVP symptoms in the afternoon. This is because symptoms commonly worsen in the morning due to prolonged fasting during the night, as gastric juice production is increased and can reflux into the esophagus, causing NVP^(2)^.

In this study, lower scores were observed in items related to feeling that NV symptoms are not part of a normal pregnancy, not being able to enjoy pregnancy, and everything being an effort when related to the occurrence of NVP. These results allow us to affirm that these women were resilient in the face of the discomfort caused by NVP, which was perceived as a physiological change inherent to pregnancy.

Although the participants were resilient in terms of physical discomfort, they reported difficulty in maintaining work, daily activities, and social activities. After the application of therapies, a decrease in these reports was observed, demonstrating that early treatment helps improve quality of life and reduces the chances of progression to hyperemesis gravidarum. Early treatment is particularly important when considering that hyperemesis gravidarum, which has a major impact on daily and social activities, causes suffering, functional incapacity, and influences thoughts about future pregnancies^(32)^.

Ferrous sulfate may also influence the presence of NVP, as this is a side effect of the medication^(33)^. However, as this supplement is prescribed to all pregnant women, as recommended by the Ministry of Health, in order to prevent iron deficiency anemia, it is believed that its use did not interfere with the results found. Thus, there was no difference in the pattern of use among women in the four groups.

Pharmacological and non-pharmacological methods can be used to alleviate the symptoms of this condition, regardless of the factors associated with NVP. As a pharmacological method, the use of antiemetics, such as metoclopramide, is a variable of interest in the present study. In Norway, a retrospective cohort observed the implications of using antiemetics during pregnancy, which were associated with fetal side effects, such as an increased risk of orofacial clefts and a tendency toward early hospitalization during pregnancy^(3)^.

Also in Norway, a study reported an association between NVP symptoms and multiparity, which could not be observed in the present study, as all groups consisted mainly of multigravidae^(34)^. The study’s findings, however, corroborate those of a national study that found that complementary therapies reduce consumption and damage caused by allopathic medications^(35)^.

Auriculotherapy is an important non-pharmacological method because, in addition to being practical and low-cost, it has been shown to reduce NVP symptoms without the use of medication^(36)^, especially when used at the onset of symptoms. This benefit can be observed in the present study, since most of the pregnant women began prenatal care at an appropriate gestational age, around the eighth week of pregnancy. Other non-pharmacological methods described in the literature that can be used in mild cases are water aerobics, vitamin B6, and ginger intake^(2,37-38)^. These therapies qualify the practice of nurses in primary care units, as they promote comprehensive care and the recovery of popular knowledge. This is a scenario in which professionals are compelled to make use of health education in their daily work with the aim of implementing less invasive and medicalized practices^(35)^, especially in the context of prenatal care.

Health education about these therapies promotes self-awareness among women and the development of the concept of health, encouraging their active participation in the health-disease process and prompting reflection on access to these therapies within the unified health system^(35)^. Thus, complementary therapies are considered mild technologies in the innovation of care and treatment of NVP and are relevant for restoring the quality of life of pregnant women. Therefore, it is suggested that these therapies be included in nursing care, as they are considered less invasive practices and are better accepted due to their positive effects^(8)^.

The use of auriculotherapy in mild cases is already recommended by FEBRASGO. However, in this study, the mean PUQE scores before intervention indicated a moderate degree of NVP, and both intervention groups showed a significant reduction in this score after 96 hours of intervention. This can be explained because auriculotherapy takes a minimum of 3 to 4 days to reach its maximum effect, with its effectiveness decreasing after this period, up to the 7th day^(37)^. No records were found in the literature on the duration of effectiveness of laser auriculotherapy; however, in the present study, the duration was similar to that of auriculotherapy.

No records were found in the literature on the effect of laser auriculotherapy for NVP. The findings of the present study, however, demonstrate that its use is as effective as the use of semi-permanent needles for the treatment of these symptoms, both in mild and moderate cases. This may favor it becoming the method of choice among non-pharmacological methods, since it is painless and has great potential for acceptance by pregnant women. The similar efficacy is probably due to the anti-inflammatory effect of vagus nerve stimulation in the auricle^(39)^.

Auricular stimulation with semi-permanent needles can reduce serum levels of C-reactive protein (CRP), tumor necrosis factor-α (TNFα), interleukin (IL)-6, and IL-10^(39-40,6)^, which are involved in the inflammatory process and pain. The signaling pathway responsible for this reduction is regulated by peripheral stimulation of the vagus nerve, which promotes an anti-inflammatory response^(39)^. Photobiomodulation caused by the use of low-power lasers, at the same wavelength used in this proposal, has been shown to be effective in anti-inflammatory signaling, with a consequent reduction in TNF-α and interleukins - IL-1β, IL-8, and IL-12^(41)^.

Despite the promising results of the study, some limitations need to be considered, such as the impossibility of blinding participants and acupuncturists and performing subgroup analyses. Furthermore, the response time of laser auriculotherapy, the degree of stress and anxiety of pregnant women at the two moments of evaluation, the adverse effects in a systematic way, and the reduced time in monitoring the permanence of the benefits obtained were not evaluated.

It is recommended that future studies be more comprehensive (multicenter) and include an assessment of the costs of applying auriculotherapy and laser auriculotherapy techniques, the acceptability of the participants, and eating habits, and that they adopt a study design that evaluates the long-term effectiveness of the intervention.

## Conclusion

The results of this study showed that auriculotherapy and laser auriculotherapy are effective non-pharmacological methods for reducing NVP and improving quality of life. Furthermore, laser auriculotherapy is as effective as auriculotherapy in reducing NVP and, as there is no discomfort in its application, it may be more acceptable to pregnant women, contributing to the use of complementary practices as non-pharmacological methods for reducing these symptoms.

The use of these therapies also contributed to a reduction in the consumption of allopathic medications by pregnant women and an improvement in quality of life, which means the possibility of resuming daily work and social activities.

## Data Availability

**All data generated or analysed during this study are included in this published article.**
