# Supplementary material for: Effectiveness of auriculotherapy and laser acupuncture in hyperemesis gravidarum: a randomized clinical trial
Source: Rev Lat Am Enfermagem. 2026 Jan 19;34:e4771. doi: 10.1590/1518-8345.7235.4771 (PMC12854655; doi:10.1590/1518-8345.7235.4771)
Supplement: Translation

# Translation: Eficácia da auriculoterapia e laserpuntura na êmese gravídica: ensaio clínico randomizado*

**DOI:** 10.1590/1518-8345.7235.4772

**Authors:** Lais de Lima Oliva, Renne Rodrigues, Keli Regiane Tomeleri da Fonseca Pinto, Sonia Silva Marcon, Catia Campaner Ferrari Bernardy

**Affiliations:** 1 Universidade Estadual de Londrina, Londrina, PR, Brasil. Universidade Estadual de Londrina PR Londrina Brasil; 2 Universidade Federal da Fronteira do Sul, Curso de Medicina, Chapecó, SC, Brasil. Universidade Federal da Fronteira do Sul Curso de Medicina SC Chapecó Brasil; 3 Universidade Estadual de Londrina, Departamento de Saúde Coletiva, Londrina, PR, Brasil. Universidade Estadual de Londrina Departamento de Saúde Coletiva PR Londrina Brasil; 4 Universidade Estadual de Londrina, Departamento de Enfermagem, Londrina, PR, Brasil. Universidade Estadual de Londrina Departamento de Enfermagem PR Londrina Brasil; 5 Universidade Estadual de Maringá, Departamento de Enfermagem, Maringá, PR, Brasil. Universidade Estadual de Maringá Departamento de Enfermagem PR Maringá Brasil

**Keywords:** Terapias Complementares, Terapia com Luz de Baixa Intensidade, Auriculoterapia, Gravidez, Êmese Gravídica, Qualidade de Vida

## Abstract

avaliar a eficácia da auriculoterapia e laserpuntura auricular em náuseas, vômitos e qualidade de vida de gestantes.

estudo randômico, paralelo, fatorial, duplo-cego, com 100 gestantes alocadas em quatro grupos (25 em cada): intervenção (G1- auriculoterapia e G2- laserpuntura auricular), controle (G3) e placebo com algodão (G4), todas acompanhadas por sete dias para análise do desfecho. Utilizaram-se o Escore de *Pregnancy Unique Quantification of Emesis* e o Grau de Palpação auricular para análise das náuseas e vômitos, e o *Health-RelatedQuality of Life Questionnaire for Nausea and Vomiting of Pregnancy* para qualidade de vida.

comparado ao grupo placebo, as participantes dos G1 e G2 reduziram (p<0,050) as pontuações do Escore *Pregnancy Unique Quantification of Emesis* em -1,14 (IC95%: -2,11; -0,17) e -1,3 (IC95%: -2,24; -0,41) e do *Health-RelatedQuality of Life Questionnaire for Nausea and Vomiting of Pregnancy* em -34,81 (IC95%: -62,98; -0,95) e -31,97 (IC95%: -62,98; -0,95), respectivamente. O uso de antieméticos foi maior nos grupos controle e placebo.

os dois tipos de intervenção demonstraram redução significativa de náuseas/vômitos e de uso de medicações alopáticas, melhora da qualidade de vida e potencial de aceitação da laserpuntura auricular pelas gestantes. Registro Brasileiro de Ensaios Clínicos RBR-4wtq84v.

## Destaques

**(1)** O uso da laserpuntura auricular é tão eficaz quanto a auriculoterapia para NVG. **(2)** A laserpuntura e auriculoterapia diminuem o uso de medicamentos alopáticos para NVG. **(3)** A laserpuntura auricular é uma opção indolor no tratamento de NVG.

## Introdução

As náuseas e vômitos são comuns no início da gestação e podem provocar efeitos negativos sobre a gravidez, dependendo de sua intensidade e frequência, bem como interferir na capacidade laboral e comprometer a qualidade de vida das mulheres neste período^(1)^. Estão presentes em 85% dos casos, porém são mais frequentes entre a 5ª e a 9ª semanas de gestação (90%), reduzindo progressivamente até tornar-se infrequentes após a 20ª semana. Os quadros que precisam de tratamento medicamentoso estão próximos de 10%, e aproximadamente 1,1% das gestações evoluem para a forma grave denominada hiperêmese gravídica^(2)^.

Diante da alta incidência de Náusea e Vômito Gestacional (NVG) e das limitações diárias recorrentes dessa condição clínica e, ainda, das possíveis implicações negativas que o uso de medicamentos durante o período gestacional pode trazer para as mulheres, as quais envolvem desde a preocupação com a saúde e formação do feto^(3)^ até questões econômicas ou mesmo efeitos colaterais na saúde física e/ou mental das mulheres acometidas, é fundamental a busca de alternativas que possibilitem uma abordagem não farmacológica, ainda que esta tenha efeito positivo na diminuição dos sintomas e consequentemente na qualidade de vida dessas mulheres. Também é importante que a alternativa proposta seja de baixo custo, fácil aceitação pelas usuárias e viável de ser utilizada nos serviços públicos de saúde, responsáveis pela assistência e acompanhamento da grande maioria das mulheres durante o período gestacional no Brasil.

Uma abordagem não farmacológica que pode auxiliar no tratamento dessas queixas é a acupuntura, a qual é, inclusive, recomendada pela Federação Brasileira das Associações de Ginecologia e Obstetrícia (FEBRASGO)^(2)^. Trata-se de uma terapia complementar caracterizada pelo estímulo, por agulhas ou laser, de pontos específicos. Quando aplicada no pavilhão auricular, denomina-se auriculoterapia^(2)^ ou laserpuntura auricular, utilizada em pessoas sensíveis ao desconforto da agulha ou com distúrbios de coagulação.

Os pontos auriculares estão ligados às demais estruturas do organismo por terminações sensoriais do nervo vago. Os neurônios vagais projetam uma extremidade para os órgãos, e a outra conecta-se ao núcleo do trato solitário no tronco encefálico, que origina os nervos cranianos, e transmitem potenciais de ação para controlar a função dos órgãos. Após um estímulo auricular, a produção de neurotransmissores que modulam o eixo cérebro-gastrointestinal é desencadeada, reduzindo a hipersensibilidade visceral e regulando a motilidade gástrica^(4-6)^, consequentemente reduzindo a manifestação de sintomas como náuseas e vômitos.

A acupuntura está prevista na Política Nacional de Práticas Integrativas e Complementares (PNPIC)^(7)^ como uma das práticas que pode ser ofertada à população em todos os níveis de assistência do Sistema Único de Saúde. Contudo, é no âmbito da Atenção Primária que ela tem sido efetivamente incorporada, sendo os enfermeiros responsáveis por um número expressivo de procedimentos^(8)^.

Os benefícios da acupuntura têm sido frequentemente relatados na literatura para diferentes problemas e públicos. Considerando mulheres durante o período gestacional, na literatura nacional encontram-se relatos do uso da acupuntura em casos de náuseas e vômitos^(9)^ e auriculoterapia para alívio de dores lombares^(10-12)^, estresse^(13)^ ansiedade^(14)^ e insônia^(15)^. No âmbito internacional, estudo de revisão que incluiu 13 Ensaios Clínicos Randomizados (ECR) considerou inconclusiva a eficácia da auriculoterapia nas náuseas e vômitos durante a gravidez, seja devido ao tamanho das amostras ou aos aspectos metodológicos adotados nos estudos^(16)^. Ainda, ECR com 128 gestantes concluiu que a auriculoterapia promoveu diminuição significativa das náuseas, mas não dos vômitos, resultado que os autores relacionaram com o fato de as participantes terem de pressionar os pontos três vezes por dia, por pelo menos 30 segundos, durante quatro dias^(17)^.

Destaca-se que não foram encontrados, na literatura disponível, ECR que avaliaram o efeito da laserpuntura auricular no controle de NVG. A ausência de evidências científicas robustas sobre a eficácia da auriculoterapia e laserpuntura auricular entre gestantes com náuseas e vômitos realça o ineditismo do estudo, cujos resultados e protocolo utilizado poderão contribuir para a prática profissional do enfermeiro. Vale lembrar que o uso de Práticas Alternativas e Complementares (PICS), que incluem a acupuntura, é reconhecido pelo Conselho Federal de Enfermagem por meio da Resolução Nº 739 de 2024^(18)^. Diante do exposto, definiu-se como objetivo avaliar a eficácia da auriculoterapia e laserpuntura auricular em náuseas, vômitos e qualidade de vida de gestantes.

## Método

### Tipo de estudo

Trata-se de um estudo clínico randomizado, paralelo, fatorial, duplo-cego, com taxa de alocação de 1:1^(19)^.

### Local de estudo

A pesquisa foi realizada em três Unidades Básicas de Saúde (UBS) de um município do norte do Paraná, selecionadas por apresentarem maior fluxo de atendimento a gestantes e oferecerem consulta de pré-natal compartilhada entre médicos e residentes em enfermagem obstétrica. Cada UBS atendia em média 30 gestantes ao mês, e as três possuíam extensão territorial semelhante.

### População e critérios de seleção

As participantes foram gestantes com risco habitual que realizavam o pré-natal nas três UBSs selecionadas. Foram incluídas gestantes com idade superior a 18 anos, idade gestacional entre 5 e 16 semanas, independentemente da paridade, que apresentaram grau leve ou moderado de sintomas de náusea e/ou vômito pelo Escore de *Pregnancy Unique Quantification of Emesis* – PUQE (Escore Leve<6; Escore Moderado entre 7 e 11). Por sua vez, não foram incluídas gestantes com deficiência mental diagnosticada e ideação suicida, distúrbios de coagulação, em tratamento farmacológico por doença psicossomática, como ansiedade e depressão, hipertireoidismo, lesão dermatológica no pavilhão auricular, febre inexplicável, gestação múltipla, doença trofoblástica gestacional, hiperêmese gravídica, uso de antiemético, diarreia, gestantes de fetos com síndrome de Down e gestantes com receio de receber a agulha semipermanente.

### Definição da amostra

O cálculo amostral foi realizado no programa *StataCorp, College Station* (Stata), versão 16.0, considerando-se os seguintes parâmetros: 4 grupos de intervenção: Poder: 0,80 (80%), alfa: 0,05, tamanho do efeito moderado das diferenças (ƞ2): 0,15 e variância do erro intragrupos: 1,0. Considerando-se os quatro grupos de intervenção, optou-se por uma medida de tamanho de efeito adequada para modelos de ANOVA (ƞ2)^(20)^. Os parâmetros empregados consideraram as recomendações para uma amostragem sistemática que permitisse que as análises realizadas fossem suficientes para detectar diferenças clinicamente relevantes, evitando erros tipo I e II^(21)^. Com base nesses parâmetros, foi determinada uma amostra mínima de 80 participantes (n=20 por grupo), e acrescidos 25% para possíveis perdas, totalizando 100 participantes (25 em cada grupo). O plano amostral seguiu as diretrizes do *Consolidated Standards of Reporting Trials* (CONSORT).

A randomização simples em quatro grupos foi realizada por um pesquisador auxiliar, utilizando-se o *website*https://www.randomizer.org e colocando-se os resultados em envelopes opacos que só foram abertos no momento do atendimento e na presença da pesquisadora acupunturista e da paciente.

### Instrumentos

O PUQE é um teste de autorrelato utilizado para avaliar a gravidade de NVG, traduzido e validado pela FEBRASGO, constituído por três questões com respostas em escala do tipo Likert de cinco pontos (Figura 1), podendo ser utilizados três tempos de corte (últimas 6, 12 ou 24 horas). O tempo mais prolongado (24 horas) é o mais utilizado, sendo o tempo inferior a 6 horas utilizado para avaliar o efeito terapêutico de alguma intervenção e para diagnóstico da intensidade de NVG^(2)^.

**Figura 1-:** Determinação da gravidade da náusea e do vômito gestacional. Escore de *Pregnancy Unique Quantification of Emesis*. Paraná, Brasil, 2023

1 – Por quanto tempo se sentiu nauseada nas últimas 24 horas?
Nunca (1) – Até 4 horas (2) – Até 8 horas (3) – Até 12 horas (4) – > de 12 horas (5)
2 – Quantos episódios de vômito apresentou nas últimas 24 horas?
Nunca (1) – Um episódio (2) – Até 3 episódios (3) – Até 4 episódios (4) – mais de cinco (5)
3 – Quantos momentos observou intensa salivação e esforço de vômito nas últimas 24 horas?
Nenhum (1) – Até 3 vezes (2) – Até 5 vezes (3) – Até 8 vezes (4) – Todo o tempo (5)
**Classificação** – Pontuação ≤ 6, forma leve; entre 7 e 11, forma moderada; ≥ 12, forma grave

Fonte: Federação Brasileira das Associações de Ginecologia e Obstetrícia^(2)^

O instrumento *Health-Related Quality of Life Questionnaire for Nausea and Vomiting of Pregnancy* (NVPQOL) é um questionário que avalia a qualidade de vida de gestantes com NVG, constituído por 30 questões distribuídas em quatro domínios: sintomas físicos e seus agravantes, exaustão, fatores emocionais e limitações. As respostas são apresentadas em escala do tipo Likert de 7 pontos, em que 1 representa ausência do sintoma referido nos últimos sete dias e 7 representa a presença do sintoma o tempo todo. A pontuação total pode variar de 30 a 210, sendo que quanto menor a pontuação, maior é a qualidade de vida^(22-23)^. A versão utilizada foi validada e traduzida para o português do Brasil em 2021 e apresentou forte consistência interna (α de Cronbach: 0,95), forte confiabilidade intra-avaliador e teste-reteste (P < 0,0; coeficiente de correlação intraclasse: 0,89; intervalo de confiança: 0,791-0,945)^(23)^.

Utilizou-se também a reatividade à palpação auricular, classificada em três níveis: Grau I – dor referida verbalmente; Grau II – dor expressa na face, piscar dos olhos ou franzimento das sobrancelhas; Grau III – paciente tenta impedir o exame retirando a cabeça ou levando sua mão até a mão do examinador. Essa classificação avalia a evolução do paciente diante do quadro de sensibilização. Espera-se que, à medida que o tratamento evolui, o paciente fique menos sensível aos estímulos do exame^(24)^. Para a palpação auricular, utilizou-se um palpador para identificar o limiar de dor à pressão. Esse instrumental possui a ponta esférica e lisa com dois milímetros de diâmetro.

Para a laserpuntura auricular, utilizou-se o aparelho *Therapy EC,* fabricado em 2018, pela empresa *Dental Manufacturing Company* (DMC), que possui dois emissores com diodo laser, sendo um vermelho e o outro infravermelho, e sistema de entrega de fibra óptica individual para cada emissor. O diâmetro útil das fibras ópticas é de 600 µm cada fibra. Conta também com um feixe de mira de baixa intensidade para indicação do ponto antecipado de impacto do laser infravermelho. A potência útil do emissor é de 100 mW ± 20%, com onda contínua e duração de pulso de 10 segundos por Joule (J). Foram utilizados óculos de proteção durante a aplicação do laser, pelas gestantes e pela pesquisadora.

Os parâmetros de irradiação utilizados foram: laser vermelho com comprimento de onda 660 nm ± 10 nm, energia de 3 J por ponto auricular, que equivalem à densidade de energia de 107 J/cm^2^ por ponto, dessa forma, irradiaram-se 7 pontos auriculares em uma única sessão, totalizando a energia acumulada de 21 J no pavilhão auricular direito. Para aplicação dos 7 pontos, foram necessários 3,5 minutos. A área do ponto auricular corresponde a aproximadamente 0,028 cm²^(25-26)^.

O grupo auriculoterapia (G1) recebeu a aplicação de uma agulha semipermanente, em aço inoxidável, esterilizada e descartável, da marca Complementar Agulhas, Registro ANVISA nº 80731150002, com dimensão de 0,20 x 1,5 mm, que ficou fixa a um micropore bege, com dimensão de 0,8 x 0,8 cm. As participantes do grupo placebo (G4) receberam a aplicação de uma esfera de algodão com menos de 1,7 mm fixada com micropore.

### Período do estudo e coleta de dados

A coleta de dados ocorreu entre abril e outubro de 2022, em sala exclusiva para os atendimentos da pesquisa nas próprias unidades.

Após cálculo amostral, as residentes em enfermagem obstétrica atuantes nas UBSs encaminharam para a acupunturista um total de 117 gestantes, para que a população estimada estatisticamente fosse alcançada. Destas, 17 não foram incluídas no estudo, 13 por indisponibilidade de tempo devido ao trabalho e quatro por dificuldade no deslocamento. As 100 participantes foram alocadas igualitária e aleatoriamente em quatro grupos (N=25 por grupo): intervenção (G1, agulha semipermanente e G2, laserpuntura auricular), G3 controle e G4 placebo. As mulheres foram acompanhadas por sete dias, para avaliação do desfecho.

A seleção das gestantes, segundo os critérios estabelecidos, foi realizada pelas residentes que explicavam a natureza da pesquisa e agendavam uma consulta com a acupunturista (enfermeira obstétrica devidamente preparada para aplicar as duas técnicas de intervenção).

No dia da consulta com a acupunturista, antes do atendimento, as residentes conduziram uma entrevista preliminar, ocasião em que também solicitaram a assinatura do Termo de Consentimento Livre e Esclarecido (TCLE). Durante a entrevista, foi utilizado um instrumento constituído de duas partes: a primeira com questões sobre características sociodemográficas e obstétricas e a segunda com dois questionários, o PUQE^(2)^ e o NVPQOL^(22-23)^. Esses instrumentos foram aplicados pelas residentes no primeiro momento e pela enfermeira da UBS no segundo (sétimo dia após o primeiro atendimento), antes do atendimento da acupunturista. Os formulários preenchidos foram encaminhados para análise pelo estatístico. Assim, residentes e estatístico foram cegados no estudo.

Todas as participantes tiveram o pavilhão auricular direito avaliado pela acupunturista, independentemente do grupo a que pertencessem. A inspeção pôde mostrar a presença de manchas, hipervascularização, escamações, nódulos ou pápulas. Após a inspeção visual, foi realizada a palpação auricular com o palpador.

O protocolo utilizado para náuseas e vômitos (NV) foi composto pelos pontos: Estômago, Cárdia, Sistema Nervoso Central, Sistema Nervoso Autônomo, Rim, Subcórtex e Occipital, determinados com base em estudos prévios e literaturas bases sobre auriculoterapia^(14,24,27-28)^. A acupunturista palpou os acupontos para identificar a sensibilidade à pressão na superfície auricular e classificou a reatividade à palpação em dois momentos: no primeiro atendimento (Palpação 1) e no retorno, sete dias depois (Palpação 2).

As agulhas semipermanentes foram aplicadas no pavilhão auricular direito das integrantes do G1, nos pontos para NV determinados no protocolo_._ Foi realizada uma única sessão, com retorno no sétimo dia. As gestantes foram orientadas a não estimular os pontos, a manter o pavilhão auricular seco, a inspecionar a integridade do micropore que fixa a agulha e observar reações, como vermelhidão local, dor, tontura, taquicardia ou sudorese, e a entrar em contato com a acupunturista imediatamente na presença de qualquer uma delas. Nesse contato, seriam orientadas a retornar para reinspeção, ajuste ou remoção da agulha semipermanente, com vistas a minimizar o viés de exclusão. Porém, nenhuma participante necessitou fazer contato com a acupunturista.

As integrantes do G2 receberam a aplicação pontual nos mesmos pontos utilizados no G1 e retornaram no sétimo dia para reavaliação. Esse grupo não teve nenhum material fixado no pavilhão auricular e não realizou nenhum estímulo no intervalo entre um atendimento e outro. As participantes do G3 foram atendidas individualmente pela acupunturista que realizou a inspeção e palpação auricular e receberam as orientações quanto ao preenchimento do escore de PUQE e retorno no sétimo dia para reavaliação.

As participantes do G4 receberam uma única aplicação de uma esfera de algodão no pavilhão auricular direito, em sete pontos aleatórios determinados pela racionalidade anatômica (joelho, tornozelo, cotovelo, perna, braço, pescoço e pulmão)^(24,27)^, com retorno no sétimo dia para reavaliação. Foram orientadas a não aplicar pressão ou manipulação sobre o local, a inspecionar a integridade do micropore e a presença de reação alérgica, como vermelhidão local. Caso apresentassem esse efeito colateral, foram orientadas a entrar em contato com a acupunturista e retornar para reinspeção, ajuste ou remoção do micropore com a esfera de algodão. As integrantes desse grupo também não precisaram fazer contato com a acupunturista.

A acupunturista e as participantes dos quatro grupos não foram cegadas em razão das diferenças físicas entre as intervenções. As participantes dos quatro grupos, além de responderem ao Escore de PUQE no primeiro atendimento (PUQE 1), receberam duas cópias do instrumento e foram orientadas a preenchê-las na 24ª (PUQE 2) e na 96ª (PUQE 3) horas, após o primeiro atendimento da acupunturista.

No segundo atendimento, sete dias depois, para classificação do grau de sensibilidade auricular, a acupunturista pressionou os mesmos pontos utilizados para NV e foram aplicados novamente o Escore de PUQE (4) e o NVPQOL (2) pela enfermeira da UBS.

A avaliação dos efeitos adversos foi realizada de forma não sistemática, e os possíveis desfechos desfavoráveis foram controlados por meio de perguntas direcionadas à presença dos possíveis sinais e sintomas ao receberem as intervenções.

Todas as participantes foram orientadas que poderiam tomar 10 mg de Metoclopramida via oral, a cada 8 horas, caso não tolerassem os sintomas de náuseas e/ou vômitos^(2)^. Uma cartela da medicação foi entregue às participantes no primeiro atendimento, e realizada contagem dos comprimidos restantes na cartela no retorno do sétimo dia.

Para evitar o viés de atrito, a acupunturista entrou em contato diariamente com as participantes, via telefone ou aplicativo de mensagens (*WhatsApp*), incluindo participantes do G3, durante o período do estudo, para garantir que o protocolo fosse seguido.

Embora algumas mulheres do G1 tenham relatado desconforto decorrente da presença das agulhas, não houve nenhuma perda, pois todas permaneceram com as agulhas até o sétimo dia. Não houve efeito adverso ao micropore que fixou as agulhas e às esferas de algodão, pois todas se mantiveram intactas durante a semana de intervenção, e as participantes não relataram sintomas de irritação na pele.

### Análise dos dados

A análise descritiva dos dados se deu por meio do número absoluto (n) e relativo (%) para as variáveis categóricas, bem como pela média e valores mínimos e máximos, ou intervalo de 95% de confiança (IC95%) para variáveis contínuas. Considerando a distribuição não normal dos dados, com base no teste de Shapiro-Wilk, utilizou-se o teste de Kruskal-Wallis (Pós-Teste: Dunn) para a comparação dos grupos em relação aos dados contínuos. Para os dados categóricos, utilizou-se o teste de Qui-Quadrado.

Adicionalmente, a análise da associação entre os grupos de intervenção e os escores das escalas PUQE e NVPQOL se deu por meio de modelos de equações de estimativa generalizada (GEE) para medidas repetidas relacionadas, com estimativa do coeficiente beta (β) e IC95%. Foram utilizados os seguintes parâmetros: distribuição normal da variável dependente, linear, matriz independente e variância robusta. Foram gerados modelos tendo como referência tanto o grupo controle como o placebo. Por fim, foram realizados modelos ajustados por possíveis fatores de confusão, tais como idade, idade gestacional, índice de massa corporal, paridade e número de consultas. A análise estatística foi realizada pelo *software Statistical Package for the Social Sciences*, adotando intervalo de confiança de 95%, e nível de significância de 5% (p<0,05) para todos os testes aplicados.

### Aspectos éticos

Este ECR foi aprovado pelo Comitê de Ética em Pesquisa Envolvendo Seres Humanos da Universidade Estadual de Londrina sob o parecer 5.091.470 e aprovado na plataforma virtual do Registro Brasileiro de Ensaios Clínicos, sob o número RBR-4wtq84v, e seguiu as recomendações do CONSORT. Os participantes foram informados sobre a pesquisa e o sigilo dos dados coletados e iniciaram a participação após assinatura do TCLE. O estudo foi financiado por recursos próprios da pesquisadora principal e da orientadora.

## Resultados

Foram avaliadas 100 gestantes quanto aos benefícios e aos danos das terapias, sem que houvesse perdas de seguimento ou respostas que pudessem comprometer a representatividade da amostra ou os resultados das análises estatísticas.

A média de idade das gestantes foi de 26,9 anos (±6,4), com mínima de 18 e máxima de 40 anos, o Índice de Massa Corporal (IMC) médio foi de 25,1 (±3,4), 81% delas tinham companheiro, 68% cursaram ensino médio completo, com renda familiar média de 3 salários-mínimos e grande parte (76%) exercia atividade remunerada. Quanto aos dados obstétricos da gestação atual, 60% eram multigestas, com idade gestacional média de 8 semanas, e 99% já haviam realizado a abertura de pré-natal e passado por pelo menos uma consulta. Não houve diferença estatística entre os grupos quanto às variáveis sociodemográficas e obstétricas (Tabela 1).

O escore de PUQE e NVPQOL antes das intervenções variaram de 8,2 a 8,7 e de 121,5 a 137,0, respectivamente, sem diferença estatística entre os grupos. Após as intervenções, houve redução estatisticamente significativa de PUQE 4 e NVPQOL 2 para os grupos auriculoterapia e laserpuntura auricular, indicando que a redução significativa dos sintomas e a melhora na qualidade de vida ocorreram após 4 dias da realização das intervenções (Tabela 2).

Antes da intervenção, a maior parte das gestantes dos quatro grupos apresentou palpação Grau II. As gestantes submetidas a auriculoterapia e a laserpuntura auricular apresentaram redução estatisticamente significativa do grau de palpação, passando para Grau I na consulta 2, significando estabilização daqueles pontos e equilíbrio do órgão correspondente. Não houve relatos de efeitos adversos nos grupos intervenção, placebo e controle.

**Tabela 1-:** Distribuição dos dados socioeconômicos, demográficos e obstétricos das gestantes com náusea e vômitos (n = 100). Paraná, Brasil, 2023

**Variáveis**	**Aurículo (G1)**	**Laser (G2)**	**Controle (G3)**	**Placebo (G4)**	**p-valor**
Idade (anos completos)*	27,1 (18; 40)	26,2 (18; 40)	28,1 (18; 38)	26,2 (18; 40)	0,688
IMC (em Kg/m²)*	24,8 (20; 34)	25,2 (21; 32)	25,6 (20; 37)	24,9 (20; 34)	0,587
Idade gestacional (semanas)*	8,4 (6; 13)	8,3 (5; 12)	7,9 (5; 13)	9,1 (5; 12)	0,319
N° de pessoas na casa*	3,2 (1; 5)	2,9 (2; 4)	2,9 (2; 6)	3,0 (2; 5)	0,378
Escolaridade ^†^					0,794
Ensino Médio incompleto ou inferior	3 (18,8)	3 (18,8)	4 (25,0)	6 (37,4)	
Ensino Médio completo	17 (25,0)	18 (26,5)	16 (23,5)	17 (25,0)	
Superior completo	5 (31,3)	4 (25,0)	5 (31,3)	2 (12,4)	
Situação conjugal ^†^					0,181
Com companheiro	17 (21,0)	20 (24,7)	23 (28,4)	21 (25,9)	
Sem companheiro	8 (42,1)	5 (26,3)	2 (10,5)	4 (21,1)	
Renda familiar (salários-mínimos) ^‡^					0,643
1	1 (11,1)	1 (11,1)	3 (33,3)	4 (44,5)	
2	2 (11,1)	5 (27,8)	5 (27,8)	6 (33,3)	
3	11 (27,5)	11 (27,5)	8 (20,0)	10 (25,0)	
4	7 (31,8)	4 (18,2)	7 (31,8)	4 (18,2)	
5 e 6	4 (36,4)	4 (36,4)	2 (18,2)	1 (9,2)	
Atividade remunerada ^†^					0,625
Sim	21 (27,6)	19 (25,0)	17 (22,4)	19 (25,0)	
Não	4 (16,7)	6 (25,0)	8 (33,3)	6 (25,0)	
Paridade ^†^					0,506
Primigesta	10 (25,0)	11 (27,5)	7 (17,5)	12 (30,0)	
Multigesta	15 (25,0)	14 (23,3)	18 (30,0)	13 (21,7)	
Número de consultas ^†^					0,640
0	0 (-)	1 (100,0)	0 (-)	0 (-)	
1	23 (26,1)	20 (22,7)	23 (26,1)	22 (25,1)	
2	2 (18,2)	4 (36,4)	2 (18,2)	3 (27,2)	

*Média (valor mínimo; valor máximo); ^†^n (%); ^‡^Salário-mínimo vigente = R$ 1.212,00, Brasil, 2022

**Tabela 2-:** Avaliação dos escores PUQE* e NVPQOL^†^ e do grau de sensibilidade à palpação auricular e do uso de medicação entre as gestantes com náusea e vômitos (n = 100). Paraná, Brasil, 2023.

**Variáveis**	**Aurículo (G1)**	**Laser (G2)**	**Controle (G3)**	**Placebo (G4)**	**p-valor**
PUQE* 1 ^‡^	8,4 (7,8; 9,1)	8,2 (7,4; 8,9)	8,7 (8,0; 9,3)	8,4 (7,8; 9,1)	0,782
PUQE* 2 ^‡^	7,8 (7,1; 8,5)	7,5 (6,7; 8,2)	8,7 (8,1; 9,4)	8,5 (7,8; 9,2)	0,042
PUQE* 3 ^‡^	6,9 (6,3; 7,5)	6,8 (6,1; 7,5)	8,5 (7,9; 9,1)	8,4 (7,8, 9,1)	<0,001
PUQE* 4 ^‡^	5,9 (5,3; 6,5)	5,8 (5,1; 6,5)	8,5 (7,9; 9,1)	8,6 (7,9; 9,2)	<0,001
NVPQOL ^†^ 1 ^‡^	121,5	127,8	134,6	137,0	0,075
	(114,0; 128,9)	(117,5; 138,2)	(126,4; 142,8)	(127,9; 146,2)	
NVPQOL ^†^ 2 ^‡^	86,0	85,8	133,4	139,6	<0,001
	(79,4; 92,7)	(77,8; 93,8)	(124,5; 142,4)	(131,1; 148,1)	
Palpação 1 ^§^					0,387
Grau I	0 (-)	0 (-)	0 (-)	1 (100,0)	
Grau II	25 (25,3)	25 (25,3)	25 (25,3)	24 (24,1)	
Palpação 2 ^§^					<0,001
Grau I	14 (43,8)	16 (50,0)	1 (3,1)	1 (3,1)	
Grau II	11 (16,2)	9 (13,2)	24 (35,3)	24 (35,3)	
Dosagem de Metoclopramida utilizada (mg)*	2,8	5,2	40,0	42,4	<0,001
	(0,6; 5,0)	(1,6; 8,8)	(28,2; 51,8)	(30,2; 54,6)	
Uso contínuo de sulfato ferroso ^§^					0,243
Sim	20 (24,1)	20 (24,1)	19 (22,9)	24 (28,9)	
Não	5 (29,4)	5 (29,4)	6 (35,3)	1 (5,9)	

*PUQE = *Pregnancy Unique Quantification of Emesis*; ^†^NVPQOL = *Health-Related Quality of Life Questionnaire for Nausea and Vomiting of Pregnancy*; ^‡^Média (intervalo de confiança de 95%); ^§^n (%)

Observou-se que as gestantes do G3 e G4 fizeram, estatisticamente, maior uso de Metoclopramida em relação aos grupos intervenção (Tabela 2). Observou-se também que a maior parte das participantes fazia uso contínuo de sulfato ferroso na dosagem de 40 mg/dia, para prevenção de anemia gestacional, sem diferença estatística entre os grupos.

Na análise da pontuação geral do escore NVPQOL antes das intervenções, houve maiores pontuações nos itens 1, 4, 8, 15 e 29, que são, respectivamente, enjoo, ânsia de vômito, sentir-se pior com certos cheiros, sentir menos interesse em sexo e dificuldade para cozinhar, apontando que os sintomas de NVG podem afetar vários âmbitos da vida da gestante e de sua família, e que o olfato pode influenciar na piora dos sintomas. Analisando os itens que apresentaram menores pontuações antes da intervenção (6, 19, 20 e 21), que são, respectivamente, a piora dos sintomas no período da tarde, sentir que seus sintomas não são de uma gravidez normal, sentir que você não pode apreciar a gravidez e tudo ser um esforço, eles indicam que, apesar dos sintomas desagradáveis, grande parte das gestantes enfrenta NV como um evento fisiológico da gestação.

Com relação à redução na pontuação após aplicação da auriculoterapia e laserpuntura auricular, os itens que mostraram maior redução foram 1, 4, 5, 8 e 9, que são, respectivamente, enjoo, ânsia de vômito, perda do apetite e sentir-se pior com certos cheiros ou comidas. A redução dos valores indica que essas terapias foram eficazes na redução desses sintomas. Dois itens apresentaram pouca redução com as intervenções, o 6 que se refere à piora dos sintomas no período da tarde, que já havia sido pouco pontuado antes da intervenção, e o item 14, sobre sentir-se emotiva, o que pode ser justificado pelas alterações hormonais ocorridas no início da gestação.

As participantes do G1 e G2 apresentaram, em modelos brutos, redução estatisticamente significativa nos escores de PUQE e NVPQOL quando comparadas às do G3 e G4 (Tabela 3). Os resultados foram, em sua maioria, replicados em modelo ajustado por fatores de confusão, com exceção do G2 para NVPQOL, que perdeu a significância estatística ao ser comparado ao G3.

Em modelo ajustado, comparando com o G4, os grupos G1 e G2 reduziram (p<0,050) as pontuações do PUQE em -1,14, com Intervalo de Confiança de 95% (IC95%: -2,11; -0,17) e -1,3 (IC95%: -2,24; -0,41), e do NVPQOL em -34,81 (IC95%: -62,98; -0,95) e -31,97 (IC95%: -62,98; -0,95) respectivamente (Tabela 3).

**Tabela 3-:** Avaliação da eficácia de auriculoterapia e laserpuntura auricular em relação aos escores PUQE* e NVPQOL^†^, entre as gestantes com náusea e vômitos (n = 100). Paraná, Brasil, 2023

**Modelo bruto**	**Controle (G3)**	**Em comparação com o grupo controle**		
		**Placebo** ^‡^ **(G4)**	**Auriculoterapia** ^‡^ **(G1)**	**Laserterapia** ^‡^ **(G2)**
PUQE*	0,00	-0,13 (-0,25; -0,01. 0,027)	-1,34 (-2,19; -0,49. 0,002)	-1,55 (-2,36; -0,74. <0,001)
NVPQOL ^†^	0,00	4,26 (1,68; 6,84. 0,001)	-30,28 (-51,01; -6,55. 0,012)	-27,22 (-55,52; 1,08. 0,059)
**Modelo ajustado** ^§^	**Controle (G3)**	**Placebo** ^‡^ **(G4)**	**Auriculoterapia** ^‡^ **(G1)**	**Laserterapia** ^‡^ **(G2)**
PUQE*	0,00	-0,26 (-0,39; -0,13. <0,001)	-1,40 (-2,25; -0,54. 0,001)	-1,59 (-2,38; -0,79. <0,001)
NVPQOL ^†^	0,00	5,99 (3,26;8,73)	-28,82 (-52,64; -4,99. 0,018)	-25,97 (-54,25; 2,31. 0,072)
**Modelo bruto**	**Placebo (G4)**	**Em comparação com o grupo placebo**		
		**Controle** ^‡^ **(G3)**	**Auriculoterapia** ^‡^ **(G1)**	**Laserterapia** ^‡^ **(G2)**
PUQE*	0,00	0,13 (0,01; 0,25. 0,027)	-1,21 (-2,18; -0,24. 0,014)	-1,42 (-2,34; -0,50. 0,002)
NVPQOL ^†^	0,00	-4,26 (-6,84; -1,68)	-34,54 (-60,84; -8,24. 0,010)	-31,48 (-62,36; -0,60. 0,046)
**Modelo ajustado** ^§^	**Placebo (G4)**	**Controle** ^‡^ **(G3)**	**Auriculoterapia** ^‡^ **(G1)**	**Laserterapia** ^‡^ **(G2)**
PUQE*	0,00	0,26 (0,13; 0,39. <0,001)	-1,14 (-2,11; -0,17. 0,022)	-1,32 (-2,24; -0,41. 0,005)
NVPQOL ^†^	0,00	-5,99 (-8,73; -3,26. <0,001)	-34,81 (-62,98; -0,95. 0,043)	-31,97 (-62,98; -0,95. 0,043)

*PUQE = *Pregnancy Unique Quantification of Emesis*; ^†^NVPQOL = *Health-Related Quality of Life Questionnaire for Nausea and Vomiting of Pregnancy*; ^‡^Beta (intervalo de confiança de 95%. p-valor); ^§^Modelo ajustado por idade, idade gestacional, índice de massa corporal, paridade e número de consultas

## Discussão

A auriculoterapia e a laserpuntura auricular foram eficazes na redução de NVG e melhoraram a qualidade de vida das gestantes. No entanto, no modelo ajustado, o G2 comparado ao G3 não apresentou melhora estatisticamente significativa na qualidade de vida, embora tenha ocorrido redução das pontuações no NVPQOL, o que nos leva a sugerir novas pesquisas com uma população maior.

A qualidade de vida das gestantes pode ser afetada pelo relacionamento com parceiro e família, os conflitos conjugais podem ser considerados fatores etiológicos de ansiedade e depressão nesse período, emoções capazes de potencializar os sintomas de NVG^(29)^. O bem-estar da gestante também pode ter relação com a vida sexual ativa nesse período, porém sintomas como NV podem influenciar negativamente na libido^(30)^, o que, no presente estudo, foi relatado por menos interesse em sexo.

Outros fatores podem intensificar a presença de NVG, como as questões hormonais, alimentação com alto teor de gorduras ou condimentados e alguns cheiros, pois o olfato é mais apurado em gestantes no primeiro trimestre. Diante disso, uma dieta saudável está associada a menor risco de desenvolver hiperêmese gravídica^(31)^.

O olfato mais aguçado em mulheres no primeiro trimestre gestacional relaciona-se ao processamento cognitivo da informação olfativa e é referido como mais alterado do que o paladar. Assim, o cheiro pode ter maior potencial para provocar os sintomas de NVG^(31)^, o que confirma os achados do presente estudo, pois as mulheres relataram que o fato de sentir certos cheiros provoca mais NV do que o fato de comer certas comidas.

Um dos itens que teve menor pontuação antes da intervenção, e praticamente não apresentou alteração após as terapias, foi a piora dos sintomas de NVG no período da tarde. Isto porque os sintomas comumente se acentuam no período da manhã, em razão do jejum prolongado que ocorre no período da noite, pois a produção de suco gástrico é aumentada e pode refluir para o esôfago, ocasionando NVG^(2)^.

Observou-se, neste estudo, menores pontuações nos itens relacionados a sentir que os sintomas de NV não são de uma gravidez normal, ao fato de não poder apreciar a gestação, e tudo ser um esforço quando relacionado à ocorrência de NVG. Esses resultados permitem afirmar que essas mulheres foram resilientes diante do desconforto causado por NVG, sendo essa situação percebida como alteração fisiológica e própria da gravidez.

Apesar de as participantes terem sido resilientes quanto aos desconfortos físicos, relataram dificuldade em manter o trabalho, as atividades diárias e atividades sociais e, após aplicação das terapias, foi observada uma diminuição desses relatos, demonstrando que o tratamento precoce auxilia na melhora da qualidade de vida e reduz as chances de evolução para um quadro de hiperêmese gravídica. O tratamento precoce é particularmente importante quando se considera que a hiperêmese gravídica, que tem grande repercussão nas atividades diárias e sociais, gera sofrimento, incapacidade funcional e influência no pensamento sobre gestações futuras^(32)^.

O sulfato ferroso também pode influenciar na presença de NVG, visto que esse é um efeito colateral da medicação^(33)^. No entanto, como esse suplemento é prescrito para todas as gestantes, conforme orientado pelo Ministério da Saúde, com o intuito de prevenir anemia ferropriva, acredita-se que seu uso não interferiu nos resultados encontrados. Destarte, não houve diferença no padrão de uso entre as mulheres dos quatro grupos.

Independentemente dos fatores associados a NVG e com a finalidade de amenizar os sintomas desse agravo, é possível utilizar métodos farmacológicos e não farmacológicos. Como método farmacológico, o uso de antieméticos, como a Metoclopramida, é variável de interesse no presente estudo. Na Noruega, uma coorte retrospectiva observou as implicações do uso de antieméticos na gestação, as quais foram associadas a efeitos colaterais fetais, como o aumento no risco de fissuras orofaciais e a tendência de hospitalização precoce na gravidez^(3).^

Ainda na Noruega, estudo relatou a associação entre os sintomas de NVG e a multiparidade, o que não pôde ser observado no presente estudo, pois todos os grupos foram constituídos, em sua maioria, por multigestas^(34)^. Os achados do estudo, no entanto, corroboram os de um estudo nacional em que foi constatado que as terapias complementares diminuem o consumo e os danos causados pelas medicações alopáticas^(35)^.

A auriculoterapia é um método não farmacológico importante, pois além de ser prático, de baixo custo, tem demonstrado ser capaz de reduzir os sintomas de NVG sem o uso de medicações^(36)^, sobretudo quando utilizado no início dos sintomas. Esse benefício pode ser observado no presente estudo, visto que grande parte das gestantes iniciaram o pré-natal em idade gestacional adequada, em torno da oitava semana gestacional. Outros métodos não farmacológicos descritos na literatura e que podem ser utilizados, nos casos leves, são hidroginástica, o uso de vitamina B6 e a ingestão do gengibre^(2,37-38)^. Essas terapias qualificam a prática do enfermeiro nas UBSs, pois promovem a integralidade do cuidado e o resgate dos conhecimentos populares. Trata-se de um cenário em que os profissionais são impelidos a fazer uso, em seu cotidiano de trabalho, da educação em saúde com o fito de implementar práticas menos invasivas e medicalizadas^(35)^, principalmente no contexto do pré-natal.

A educação em saúde sobre essas terapias promove o autoconhecimento por parte das mulheres e a elaboração do conceito de saúde, atribuindo participação ativa delas no processo saúde-doença e despertando a reflexão sobre o acesso a essas terapias no ambiente do sistema único de saúde^(35)^. Destarte, as terapias complementares são consideradas tecnologias leves na inovação do cuidado e no tratamento de NVG e mostram-se relevantes para o reestabelecimento da qualidade de vida das gestantes. Portanto, sugere-se a inserção dessas terapias no cuidado da enfermagem, visto que são consideradas práticas menos invasivas, além de serem melhor aceitas por seus efeitos positivos^(8)^.

O uso da auriculoterapia em casos leves já é recomendado pela FEBRASGO; no entanto, neste estudo, os valores médios do escore de PUQE antes da intervenção indicavam um grau moderado de NVG, e os dois grupos de intervenção apresentaram redução significativa dessa pontuação após 96 horas de intervenção. Isso pode ser explicado porque a auriculoterapia tem um tempo mínimo entre 3 e 4 dias para atingir o pico máximo de sua ação, reduzindo sua efetividade após esse período, até o 7º dia^(37)^. Não foram encontrados registros na literatura sobre tempo de eficácia da laserpuntura auricular porém, no presente estudo, o tempo foi semelhante ao da auriculoterapia.

Não foram encontrados registros na literatura sobre efeito do uso da laserpuntura auricular para NVG. Os achados do presente estudo, no entanto, demonstram que seu uso é tão eficaz quanto o uso das agulhas semipermanentes para o tratamento desses sintomas, tanto nos casos leves como nos moderados. Isso pode favorecer para que se torne opção de escolha entre os métodos não farmacológicos, visto que, por ser indolor, tem grande potencial de aceitação pelas gestantes. A eficácia semelhante deve-se provavelmente ao efeito anti-inflamatório da estimulação do nervo vago no pavilhão auricular^(39)^.

Estímulos auriculares com agulhas semipermanentes são capazes de reduzir níveis séricos de proteína C-reativa (PCR), fator de necrose tumoral-α (TNFα), interleucina (IL)-6 e IL-10^(6,39-40)^, envolvidos no processo inflamatório e dor. A via de sinalização responsável por essa redução regula-se pela estimulação periférica do nervo vago, que promove a resposta anti-inflamatória^(39)^. A fotobiomodulação causada pela utilização do laser de baixa potência, no mesmo comprimento de onda utilizado nessa proposta, demonstra eficácia na sinalização anti-inflamatória com consequente redução de TNF-α e interleucinas - IL-1β, IL-8 e IL-12^(41)^.

A despeito dos resultados promissores do estudo, algumas limitações precisam ser consideradas, tais como: a impossibilidade de cegar participantes e acupunturista e de realizar análise de subgrupos. Ainda, o fato de não ter sido avaliado o tempo resposta da laserpuntura auricular, o grau de estresse e ansiedade das gestantes nos dois momentos da avaliação, os efeitos adversos de forma sistemática, e o tempo reduzido no acompanhamento da permanência dos benefícios obtidos.

É recomendável que estudos futuros sejam mais amplos (multicêntricos) e incluam a avaliação dos custos na aplicação das técnicas de auriculoterapia e laserpuntura auricular, a aceitabilidade das participantes, os hábitos alimentares, e que adotem um desenho de estudo que avalie a eficácia da intervenção a longo prazo.

## Conclusão

Os resultados do estudo mostraram que a auriculoterapia e laserpuntura auricular são métodos não farmacológicos eficazes na redução de NVG e na melhoria da qualidade de vida. Ainda que a laserpuntura auricular é tão eficaz quanto a auriculoterapia na redução de NVG e, por não haver desconforto em sua aplicação, pode vir a ter maior aceitabilidade pelas gestantes, contribuindo para a utilização das práticas complementares como métodos não farmacológicos para redução desses sintomas.

O uso dessas terapias também contribuiu para a redução do consumo de medicamentos alopáticos pelas gestantes e a melhora da qualidade de vida, o que significa possibilidade de restabelecimento das atividades laborais diárias e sociais.

## Data Availability

Todos os dados gerados ou analisados durante este estudo estão incluídos neste artigo publicado.

# Translation: Eficacia de la auriculoterapia y la acupuntura con láser en la hiperémesis gravídica: ensayo clínico aleatorizado*

**DOI:** 10.1590/1518-8345.7235.4770

**Authors:** Lais de Lima Oliva, Renne Rodrigues, Keli Regiane Tomeleri da Fonseca Pinto, Sonia Silva Marcon, Catia Campaner Ferrari Bernardy

**Affiliations:** 1 Universidade Estadual de Londrina, Londrina, PR, Brasil. Universidade Estadual de Londrina PR Londrina Brasil; 2 Universidade Federal da Fronteira do Sul, Curso de Medicina, Chapecó, SC, Brasil. Universidade Federal da Fronteira do Sul Curso de Medicina SC Chapecó Brasil; 3 Universidade Estadual de Londrina, Departamento de Saúde Coletiva, Londrina, PR, Brasil. Universidade Estadual de Londrina Departamento de Saúde Coletiva PR Londrina Brasil; 4 Universidade Estadual de Londrina, Departamento de Enfermagem, Londrina, PR, Brasil. Universidade Estadual de Londrina Departamento de Enfermagem PR Londrina Brasil; 5 Universidade Estadual de Maringá, Departamento de Enfermagem, Maringá, PR, Brasil. Universidade Estadual de Maringá Departamento de Enfermagem PR Maringá Brasil

**Keywords:** Terapias Complementarias, Terapia por Luz de Baja Intensidad, Auriculoterapia, Embarazo, Náuseas Matinales, Calidad de Vida

## Abstract

evaluar la eficacia de la auriculoterapia y de la laserpuntura auricular en las náuseas, vómitos y calidad de vida de mujeres embarazadas.

estudio aleatorizado, paralelo, factorial y doble ciego, con 100 mujeres embarazadas asignadas a cuatro grupos (25 en cada uno): intervención (G1 – auriculoterapia y G2 – laserpuntura auricular), control (G3) y placebo con algodón (G4). Todas fueron seguidas durante siete días para el análisis del desenlace. Se utilizaron el *Pregnancy-Unique Quantification of Emesis* (PUQE) y el Grado de Palpación Auricular para analizar las náuseas y vómitos, y el *Health-Related Quality of Life Questionnaire for Nausea and Vomiting of Pregnancy* para evaluar la calidad de vida.

en comparación con el grupo placebo, las participantes de G1 y G2 redujeron (p < 0,050) las puntuaciones del PUQE en -1,14 (IC95%: -2,11; -0,17) y -1,3 (IC95%: -2,24; -0,41), y las del *Health-Related Quality of Life Questionnaire for Nausea and Vomiting of Pregnancy* en -34,81 (IC95%: -62,98; -0,95) y -31,97 (IC95%: -62,98; -0,95), respectivamente. El uso de antieméticos fue mayor en los grupos control y placebo.

ambos tipos de intervención demostraron una reducción significativa de las náuseas y vómitos, así como del uso de medicamentos alopáticos, con mejora de la calidad de vida y potencial aceptación de la laserpuntura auricular por parte de las mujeres embarazadas. Registro Brasileño de Ensayos Clínicos: RBR-4wtq84v.

## Destacados

**(1)** El uso de la laserpuntura auricular es tan eficaz como la auriculoterapia para NVE. **(2)** La laserpuntura auricular y la auriculoterapia reducen el uso de medicamentos alopáticos para NVE. **(3)** La laserpuntura auricular es una opción indolora en el tratamiento de NVE.

## Introducción

Las náuseas y los vómitos son comunes al inicio del embarazo y pueden tener efectos negativos sobre la gestación, dependiendo de su intensidad y frecuencia, además de interferir en la capacidad laboral y comprometer la calidad de vida de las mujeres en este período^(1)^. Están presentes en el 85% de los casos, aunque son más frecuentes entre la 5ª y la 9ª semana de embarazo (90%), disminuyendo progresivamente hasta volverse infrecuentes después de la 20ª semana. Los cuadros que requieren tratamiento farmacológico representan cerca del 10%, y aproximadamente el 1,1% de los embarazos evolucionan hacia la forma grave denominada hiperémesis gravídica^(2)^.

Ante la alta incidencia de Náuseas y Vómitos en el Embarazo (NVE), las limitaciones cotidianas recurrentes de esta condición clínica y, además, las posibles implicaciones negativas que el uso de medicamentos durante el embarazo puede ocasionar en las mujeres —que van desde la preocupación por la salud y el desarrollo del feto^(3)^ hasta cuestiones económicas o incluso efectos secundarios en la salud física y/o mental de las mujeres afectadas—, resulta fundamental la búsqueda de alternativas que permitan un abordaje no farmacológico, siempre que este contribuya a la disminución de los síntomas y, en consecuencia, a la mejora de la calidad de vida de estas mujeres. También es importante que la alternativa propuesta sea de bajo costo, de fácil aceptación por parte de las usuarias y viable para implementarse en los servicios públicos de salud, responsables de la atención y el acompañamiento de la gran mayoría de mujeres embarazadas durante el período gestacional en Brasil.

Una alternativa no farmacológica que puede contribuir al tratamiento de estas molestias es la acupuntura, la cual incluso está recomendada por la Federación Brasileña de Asociaciones de Ginecología y Obstetricia (FEBRASGO)^(2)^. Se trata de una terapia complementaria caracterizada por la estimulación, mediante agujas o láser, de puntos específicos. Cuando se aplica en el pabellón auricular, se denomina auriculoterapia^(2)^ o laserpuntura auricular, utilizada en personas sensibles al malestar de la aguja o con trastornos de coagulación.

Los puntos auriculares están conectados con otras estructuras del organismo a través de terminaciones sensoriales del nervio vago. Las neuronas vagales proyectan un extremo hacia los órganos y el otro se conecta con el núcleo del tracto solitario en el tronco encefálico, origen de los nervios craneales, transmitiendo potenciales de acción que regulan la función de los órganos. Tras un estímulo auricular, se desencadena la producción de neurotransmisores que modulan el eje cerebro-gastrointestinal, lo cual reduce la hipersensibilidad visceral y regula la motilidad gástrica^(4-6)^, disminuyendo así la manifestación de síntomas como náuseas y vómitos.

La acupuntura está prevista en la Política Nacional de Prácticas Integrativas y Complementarias (PNPIC)^(7)^ como una de las terapias que pueden ofrecerse a la población en todos los niveles de atención del Sistema Único de Salud. No obstante, es en el ámbito de la Atención Primaria donde ha sido efectivamente incorporada, siendo las enfermeras responsables de un número significativo de procedimientos^(8)^.

Los beneficios de la acupuntura han sido frecuentemente reportados en la literatura para diferentes problemas y poblaciones. En relación con las mujeres durante el embarazo, en la literatura nacional se encuentran reportes del uso de la acupuntura en casos de náuseas y vómitos^(9)^ así como de la auriculoterapia para el alivio del dolor lumbar^(10-12)^, estrés^(13)^, la ansiedad^(14)^ y el insomnio^(15)^. A nivel internacional, una revisión que incluyó 13 Ensayos Clínicos Aleatorizados (ECA) consideró inconclusiva la eficacia de la auriculoterapia en las náuseas y vómitos durante el embarazo, debido al tamaño de las muestras o a las limitaciones metodológicas de los estudios^(16)^. Además, un ECA con 128 mujeres embarazadas concluyó que la auriculoterapia promovió una disminución significativa de las náuseas, pero no de los vómitos, resultado que los autores atribuyeron a la necesidad de que las participantes presionaran los puntos tres veces al día, durante al menos 30 segundos, por cuatro días^(17)^.

Cabe destacar que no se encontraron en la literatura disponible ECAs que evaluaran el efecto de la laserpuntura auricular en el control de las NVE. La ausencia de evidencias científicas sólidas sobre la eficacia de la auriculoterapia y la laserpuntura auricular en mujeres embarazadas con náuseas y vómitos refuerza el carácter inédito de este estudio, cuyos resultados y protocolo utilizado podrán contribuir a la práctica profesional de enfermería. Vale recordar que el uso de Prácticas Integrativas y Complementarias en Salud (PICS), entre las que se incluye la acupuntura, está reconocido por el Consejo Federal de Enfermería a través de la Resolución Nº 739 de 2024^(18)^. A la luz de lo expuesto, se definió como objetivo evaluar la eficacia de la auriculoterapia y de la laserpuntura auricular en las náuseas, los vómitos y la calidad de vida de mujeres embarazadas.

## Método

### Tipo de estudio

Se trata de un ensayo clínico aleatorizado, paralelo, factorial y doble ciego, con una tasa de asignación de 1:1^(19)^.

### Lugar del estudio

La investigación se llevó a cabo en tres Unidades Básicas de Salud (UBS) de un municipio del norte de Paraná, seleccionadas por presentar mayor flujo de atención a mujeres embarazadas y por ofrecer consulta prenatal compartida entre médicos y residentes en enfermería obstétrica. Cada UBS atendía en promedio a 30 mujeres embarazadas por mes, y las tres tenían extensión territorial semejante.

### Población y criterios de selección

Las participantes fueron mujeres embarazadas con riesgo obstétrico habitual que realizaban el control prenatal en las tres UBS seleccionadas. Se incluyeron mujeres mayores de 18 años, con edad gestacional entre 5 y 16 semanas, independientemente de la paridad, que presentaban síntomas leves o moderados de náuseas y/o vómitos según el *Pregnancy-Unique Quantification of Emesis* (PUQE) (leve < 6; moderado entre 7 y 11). Por su parte, se excluyeron mujeres embarazadas con discapacidad intelectual diagnosticada y con ideación suicida, trastornos de coagulación, en tratamiento farmacológico por enfermedad psicosomática como ansiedad o depresión, hipertiroidismo, lesión dermatológica en el pabellón auricular, fiebre inexplicable, embarazo múltiple, enfermedad trofoblástica gestacional, hiperémesis gravídica, uso de antieméticos, diarrea, embarazo de fetos con síndrome de Down y aquellas con temor a recibir la aguja semipermanente.

### Definición de la muestra

El cálculo del tamaño muestral se realizó con el programa *StataCorp*, *College Station* (Stata), versión 16.0, considerando los siguientes parámetros: 4 grupos de intervención; poder: 0,80 (80%); alfa: 0,05; tamaño del efecto moderado de las diferencias (ƞ2): 0,15; y varianza del error intragrupal: 1,0. Teniendo en cuenta los cuatro grupos de intervención, se optó por una medida de tamaño del efecto adecuada para modelos de ANOVA (ƞ2)^(20)^. Los parámetros empleados siguieron las recomendaciones para un muestreo sistemático que permitiera realizar análisis suficientes para detectar diferencias clínicamente relevantes, evitando errores tipo I y II^(21)^. Con base en estos parámetros, se determinó un tamaño mínimo de muestra de 80 participantes (n = 20 por grupo), al que se añadieron 25% para posibles pérdidas, totalizando 100 participantes (25 en cada grupo). El plan de muestreo siguió las directrices del *Consolidated Standards of Reporting Trials* (CONSORT).

La asignación aleatoria simple en cuatro grupos fue realizada por un investigador auxiliar, utilizando el sitio web https://www.randomizer.org, y los resultados se colocaron en sobres opacos que solo fueron abiertos en el momento de la atención, en presencia de la investigadora acupunturista y de la paciente.

### Instrumentos

El PUQE es un cuestionario de autorreporte utilizado para evaluar la gravedad de las NVE, traducido y validado por la Federación Brasileña de Asociaciones de Ginecología y Obstetricia (FEBRASGO). Consta de tres preguntas con respuestas en escala tipo Likert de cinco puntos (Figura 1), pudiéndose aplicar en tres periodos de referencia (últimas 6, 12 o 24 horas). El periodo más prolongado (24 horas) es el más utilizado, mientras que el inferior a 6 horas se emplea para evaluar el efecto terapéutico de alguna intervención y para el diagnóstico de la intensidad de las NVE^(2)^.

**Figura 1-:** Determinación de la gravedad de las náuseas y vómitos en el embarazo. Puntuación de la *Pregnancy Unique Quantification of Emesis*. Paraná, Brasil, 2023

1 – ¿Durante cuánto tiempo se sintió con náuseas en las últimas 24 horas?
Nunca (1) – Hasta 4 horas (2) – Hasta 8 horas (3) – Hasta 12 horas (4) – Más de 12 horas (5)
2 – ¿Cuántos episodios de vómito presentó en las últimas 24 horas?
Ninguno (1) – Un episodio (2) – Hasta 3 episodios (3) – Hasta 4 episodios (4) – Más de 5 (5)
3 – ¿Cuántos momentos de intensa salivación y esfuerzo de vómito observó en las últimas 24 horas?
Ninguno (1) – Hasta 3 veces (2) – Hasta 5 veces (3) – Hasta 8 veces (4) – Todo el tiempo (5)
**Clasificación:** puntuación ≤ 6, forma leve; entre 7 y 11, forma moderada; ≥ 12, forma grave

Fuente: Federación Brasileña de Asociaciones de Ginecología y Obstetricia^(2)^

El *Health-Related Quality of Life Questionnaire for Nausea and Vomiting of Pregnancy* (NVPQOL) es un cuestionario que evalúa la calidad de vida de mujeres embarazadas con NVE. Está compuesto por 30 preguntas distribuidas en cuatro dominios: síntomas físicos y sus agravantes, agotamiento, factores emocionales y limitaciones. Las respuestas se presentan en una escala tipo Likert de 7 puntos, en la que 1 representa ausencia del síntoma referido en los últimos siete días y 7 representa la presencia del síntoma todo el tiempo. La puntuación total puede variar entre 30 y 210, de modo que cuanto menor sea la puntuación, mayor es la calidad de vida^(22-23)^. La versión utilizada fue traducida y validada al portugués de Brasil en 2021 y mostró una fuerte consistencia interna (α de Cronbach: 0,95), así como alta confiabilidad intraevaluador y en el test-retest (p < 0,01; coeficiente de correlación intraclase: 0,89; intervalo de confianza: 0,791-0,945)^(23)^.

También se utilizó la reactividad a la palpación auricular, clasificada en tres niveles: Grado I – dolor referido verbalmente; Grado II – dolor expresado en el rostro, parpadeo o fruncimiento de las cejas; Grado III – la paciente intenta impedir el examen moviendo la cabeza o llevando su mano hacia la mano del examinador. Esta clasificación evalúa la evolución de la paciente frente al cuadro de sensibilización. Se espera que, a medida que el tratamiento avanza, la paciente se vuelva menos sensible a los estímulos del examen^(24)^. Para la palpación auricular, se utilizó un palpador para identificar el umbral de dolor a la presión. Este instrumento tiene una punta esférica y lisa de dos milímetros de diámetro.

Para la laserpuntura auricular se empleó el dispositivo *Therapy EC*, fabricado en 2018 por la empresa *Dental Manufacturing Company* (DMC). Este equipo cuenta con dos emisores de diodo láser, uno rojo y otro infrarrojo, y un sistema de entrega de fibra óptica individual para cada emisor. El diámetro útil de las fibras ópticas es de 600 µm cada una. Además, posee un haz de guía de baja intensidad que indica el punto anticipado de impacto del láser infrarrojo. La potencia útil del emisor es de 100 mW ± 20%, con onda continua y duración de pulso de 10 segundos por Joule (J). Durante la aplicación del láser se utilizaron gafas de protección tanto por parte de las mujeres embarazadas como de la investigadora.

Los parámetros de irradiación utilizados fueron: láser rojo con longitud de onda de 660 nm ± 10 nm, energía de 3 J por punto auricular, equivalente a una densidad de energía de 107 J/cm² por punto. De este modo, se irradiaron 7 puntos auriculares en una única sesión, con una energía acumulada total de 21 J en el pabellón auricular derecho. Para la aplicación en los 7 puntos se requirieron 3,5 minutos. El área de cada punto auricular corresponde a aproximadamente 0,028 cm²^(25-26)^.

El grupo de auriculoterapia (G1) recibió la aplicación de una aguja semipermanente de acero inoxidable, estéril y desechable, de la marca *Complementar Agulhas*, Registro ANVISA nº 80731150002, con dimensiones de 0,20 x 1,5 mm, fijada con un micropore color beige de 0,8 x 0,8 cm. Las participantes del grupo placebo (G4) recibieron la aplicación de una esfera de algodón de menos de 1,7 mm, también fijada con micropore.

### Período del estudio y recolección de datos

La recolección de datos se llevó a cabo entre abril y octubre de 2022, en una sala exclusiva para las atenciones de la investigación dentro de las propias unidades.

Tras el cálculo muestral, las residentes en enfermería obstétrica que actuaban en las UBS remitieron a la acupunturista un total de 117 mujeres embarazadas para alcanzar la población estimada estadísticamente. De ellas, 17 no fueron incluidas en el estudio: 13 por falta de disponibilidad de tiempo debido al trabajo y 4 por dificultades de desplazamiento. Las 100 participantes fueron asignadas de manera equitativa y aleatoria a cuatro grupos (N = 25 por grupo): intervención (G1, aguja semipermanente; G2, laserpuntura auricular), G3 control y G4 placebo. Las mujeres fueron seguidas durante siete días para la evaluación del desenlace.

La selección de las mujeres embarazadas, de acuerdo con los criterios establecidos, fue realizada por las residentes, quienes explicaban la naturaleza de la investigación y agendaban una consulta con la acupunturista (enfermera obstétrica debidamente capacitada para aplicar ambas técnicas de intervención).

El día de la consulta con la acupunturista, antes de la atención, las residentes realizaron una entrevista preliminar, ocasión en la que también solicitaron la firma del Consentimiento Libre e Informado (CLI). Durante la entrevista, se utilizó un instrumento compuesto por dos partes: la primera con preguntas sobre características sociodemográficas y obstétricas, y la segunda con dos cuestionarios, el PUQE^(2)^ y el NVPQOL^(22-23)^. Estos instrumentos fueron aplicados por las residentes en el primer momento y por la enfermera de la UBS en el segundo (séptimo día después de la primera atención), antes de la consulta con la acupunturista. Los formularios completados fueron enviados para análisis al estadístico. De esta manera, tanto las residentes como el estadístico permanecieron cegados en el estudio.

Todas las participantes tuvieron el pabellón auricular derecho evaluado por la acupunturista, independientemente del grupo al que pertenecieran. La inspección podía mostrar la presencia de manchas, hipervascularización, descamaciones, nódulos o pápulas. Tras la inspección visual, se realizó la palpación auricular con el palpador.

El protocolo utilizado para náuseas y vómitos (NV) estuvo compuesto por los siguientes puntos: Estómago, Cardias, Sistema Nervioso Central, Sistema Nervioso Autónomo, Riñón, Subcórtex y Occipital, determinados con base en estudios previos y en la literatura de referencia sobre auriculoterapia^(14,24,27-28)^. La acupunturista palpó los acupuntos para identificar la sensibilidad a la presión en la superficie auricular y clasificó la reactividad a la palpación en dos momentos: en la primera atención (Palpación 1) y en la visita de retorno, siete días después (Palpación 2).

Las agujas semipermanentes fueron aplicadas en el pabellón auricular derecho de las integrantes del G1, en los puntos para NV determinados en el protocolo. Se realizó una única sesión, con retorno al séptimo día. Las mujeres embarazadas fueron orientadas a no estimular los puntos, mantener seco el pabellón auricular, inspeccionar la integridad del micropore que fijaba la aguja y observar reacciones como enrojecimiento local, dolor, mareo, taquicardia o sudoración, y contactar de inmediato a la acupunturista ante la presencia de cualquiera de ellas. En ese caso, serían orientadas a regresar para reinspección, ajuste o remoción de la aguja semipermanente, con el fin de minimizar el sesgo de exclusión. Sin embargo, ninguna participante necesitó contactar a la acupunturista.

Las integrantes del G2 recibieron la aplicación puntual en los mismos puntos utilizados en el G1 y regresaron al séptimo día para la reevaluación. Este grupo no tuvo ningún material fijado en el pabellón auricular y no realizó ningún tipo de estímulo en el intervalo entre una atención y otra. Las participantes del G3 fueron atendidas individualmente por la acupunturista, quien realizó la inspección y palpación auricular, y recibieron las orientaciones respecto al llenado del cuestionario PUQE y al retorno en el séptimo día para la reevaluación.

Las participantes del G4 recibieron una única aplicación de una esfera de algodón en el pabellón auricular derecho, en siete puntos aleatorios determinados según la racionalidad anatómica (rodilla, tobillo, codo, pierna, brazo, cuello y pulmón)^(24,27)^, con retorno al séptimo día para la reevaluación. Se les indicó no aplicar presión ni manipulación sobre el lugar, inspeccionar la integridad del micropore y observar posibles reacciones alérgicas como enrojecimiento local. En caso de presentar este efecto colateral, fueron orientadas a contactar a la acupunturista y regresar para reinspección, ajuste o retiro del micropore con la esfera de algodón. Ninguna de las integrantes de este grupo necesitó ponerse en contacto con la acupunturista.

La acupunturista y las participantes de los cuatro grupos no fueron cegadas debido a las diferencias físicas entre las intervenciones. Las participantes de los cuatro grupos, además de responder al cuestionario PUQE en la primera atención (PUQE 1), recibieron dos copias adicionales del instrumento y fueron orientadas a completarlas en la 24ª hora (PUQE 2) y en la 96ª hora (PUQE 3) después de la primera consulta con la acupunturista.

En la segunda atención, siete días después, para la clasificación del grado de sensibilidad auricular, la acupunturista presionó los mismos puntos utilizados para NV y nuevamente se aplicaron el cuestionario PUQE (4) y el NVPQOL (2), esta vez por la enfermera de la UBS.

La evaluación de los efectos adversos se realizó de manera no sistemática, y los posibles desenlaces desfavorables fueron controlados mediante preguntas dirigidas a la presencia de signos y síntomas al momento de recibir las intervenciones.

Todas las participantes fueron orientadas a que podían tomar 10 mg de metoclopramida por vía oral cada 8 horas, en caso de no tolerar los síntomas de náuseas y/o vómitos^(2)^. En la primera atención se entregó un blíster de la medicación a cada participante, y en la visita de retorno al séptimo día se realizó el conteo de los comprimidos restantes.

Para evita el sesgo por deserción, la acupunturista se puso en contacto diariamente con las participantes, incluyendo las del G3, a través de llamadas telefónicas o mediante la aplicación de mensajería *WhatsApp*, durante todo el período del estudio, con el fin de asegurar que se cumpliera el protocolo.

Aunque algunas mujeres del G1 reportaron molestias derivadas de la presencia de las agujas, no hubo pérdidas, ya que todas permanecieron con las agujas hasta el séptimo día. No se registraron efectos adversos relacionados con el micropore que fijaba las agujas o las esferas de algodón, ya que todos se mantuvieron intactos durante la semana de intervención, y las participantes no informaron síntomas de irritación cutánea.

### Análisis de datos

El análisis descriptivo de los datos se realizó mediante número absoluto (n) y relativo (%) para las variables categóricas, así como a través de la media y valores mínimos y máximos o intervalo de confianza del 95% (IC95%) para las variables continuas. Considerando la distribución no normal de los datos, con base en la prueba de Shapiro-Wilk, se empleó la prueba de Kruskal-Wallis (*post hoc*: Dunn) para la comparación de los grupos en relación con los datos continuos. Para los datos categóricos, se utilizó la prueba de Chi-cuadrado.

Adicionalmente, el análisis de la asociación entre los grupos de intervención y los puntajes de las escalas PUQE y NVPQOL se llevó a cabo mediante modelos de ecuaciones de estimación generalizada (GEE) para medidas repetidas relacionadas, con estimación del coeficiente beta (β) y IC95%. Se emplearon los siguientes parámetros: distribución normal de la variable dependiente, modelo lineal, matriz independiente y varianza robusta. Se generaron modelos considerando como referencia tanto el grupo control como el placebo. Finalmente, se realizaron modelos ajustados por posibles factores de confusión, tales como edad, edad gestacional, índice de masa corporal, paridad y número de consultas. El análisis estadístico se realizó con el software *Statistical Package for the Social Sciences* (SPSS), adoptando un intervalo de confianza del 95% y un nivel de significancia del 5% (p < 0,05) para todas las pruebas aplicadas.

### Aspectos éticos

Este ECA fue aprobado por el Comité de Ética en Investigación con Seres Humanos de la Universidad Estadual de Londrina bajo el dictamen nº 5.091.470 y registrado en la plataforma virtual del Registro Brasileño de Ensayos Clínicos con el número RBR-4wtq84v, siguiendo las recomendaciones del CONSORT. Las participantes fueron informadas sobre la investigación y la confidencialidad de los datos recolectados, e iniciaron su participación tras la firma del Consentimiento Libre e Informado (CLI).

## Resultados

Se evaluaron 100 mujeres embarazadas en cuanto a los beneficios y riesgos de las terapias, sin que hubiera pérdidas de seguimiento ni respuestas que pudieran comprometer la representatividad de la muestra o los resultados de los análisis estadísticos.

La edad promedio de las mujeres embarazadas fue de 26,9 años (±6,4), con mínima de 18 y máxima de 40 años; el Índice de Masa Corporal (IMC) promedio fue de 25,1 (±3,4); el 81% tenía pareja; el 68% había cursado la educación secundaria completa; el ingreso familiar promedio fue de tres salarios mínimos; y la mayoría (76%) ejercía actividad remunerada. En relación con los datos obstétricos del embarazo actual, el 60% eran multíparas, con edad gestacional promedio de 8 semanas, y el 99% ya había iniciado el control prenatal y asistido al menos a una consulta. No se encontraron diferencias estadísticas entre los grupos en cuanto a las variables sociodemográficas y obstétricas (Tabla 1).

**Tabla 1-:** Distribución de los datos socioeconómicos, demográficos y obstétricos de las embarazadas con náuseas y vómitos (n = 100). Paraná, Brasil, 2023

**Variables**	**Auriculoterapia (G1)**	**Láser (G2)**	**Control (G3)**	**Placebo (G4)**	**Valor p**
Edad (años completos)*	27,1 (18; 40)	26,2 (18; 40)	28,1 (18; 38)	26,2 (18; 40)	0,688
IMC (en Kg/m²)*	24,8 (20; 34)	25,2 (21; 32)	25,6 (20; 37)	24,9 (20; 34)	0,587
Edad gestacional (semanas)*	8,4 (6; 13)	8,3 (5; 12)	7,9 (5; 13)	9,1 (5; 12)	0,319
Número de personas en el hogar*	3,2 (1; 5)	2,9 (2; 4)	2,9 (2; 6)	3,0 (2; 5)	0,378
Nivel educativo ^†^					0,794
Secundaria incompleta o inferior	3 (18,8)	3 (18,8)	4 (25,0)	6 (37,4)	
Secundaria completa	17 (25,0)	18 (26,5)	16 (23,5)	17 (25,0)	
Superior completa	5 (31,3)	4 (25,0)	5 (31,3)	2 (12,4)	
Situación marital ^†^					0,181
Con pareja	17 (21,0)	20 (24,7)	23 (28,4)	21 (25,9)	
Sin pareja	8 (42,1)	5 (26,3)	2 (10,5)	4 (21,1)	
Ingresos familiares (salarios mínimos) ^‡^					0,643
1	1 (11,1)	1 (11,1)	3 (33,3)	4 (44,5)	
2	2 (11,1)	5 (27,8)	5 (27,8)	6 (33,3)	
3	11 (27,5)	11 (27,5)	8 (20,0)	10 (25,0)	
4	7 (31,8)	4 (18,2)	7 (31,8)	4 (18,2)	
5 y 6	4 (36,4)	4 (36,4)	2 (18,2)	1 (9,2)	
Actividad remunerada ^†^					0,625
Sí	21 (27,6)	19 (25,0)	17 (22,4)	19 (25,0)	
No	4 (16,7)	6 (25,0)	8 (33,3)	6 (25,0)	
Paridad ^†^					0,506
Primigesta	10 (25,0)	11 (27,5)	7 (17,5)	12 (30,0)	
Multigesta	15 (25,0)	14 (23,3)	18 (30,0)	13 (21,7)	
Número de consultas ^†^					0,640
0	0 (-)	1 (100,0)	0 (-)	0 (-)	
1	23 (26,1)	20 (22,7)	23 (26,1)	22 (25,1)	
2	2 (18,2)	4 (36,4)	2 (18,2)	3 (27,2)	

*Media (valor mínimo; valor máximo); **†**n (%); **‡**Salario mínimo vigente = R$ 1.212,00, Brasil, 2022

Los puntajes de PUQE y NVPQOL antes de las intervenciones variaron entre 8,2 y 8,7 y entre 121,5 y 137,0, respectivamente, sin diferencias estadísticas entre los grupos. Después de las intervenciones, se observó una reducción estadísticamente significativa en PUQE 4 y NVPQOL 2 para los grupos de auriculoterapia y de laserpuntura auricular, lo que indica que la disminución significativa de los síntomas y la mejora en la calidad de vida ocurrieron a los 4 días de realizadas las intervenciones (Tabla 2).

Antes de la intervención, la mayoría de las mujeres embarazadas de los cuatro grupos presentaban palpación de Grado II. Las mujeres embarazadas sometidas a auriculoterapia y a laserpuntura auricular mostraron una reducción estadísticamente significativa en el grado de palpación, pasando a Grado I en la consulta 2, lo que indica estabilización de esos puntos y equilibrio del órgano correspondiente. No se registraron efectos adversos en los grupos de intervención, placebo y control.

**Tabla 2-:** Evaluación de las puntuaciones PUQE* y NVPQOL^†^, así como del grado de sensibilidad a la palpación auricular y del uso de medicamentos entre las embarazadas con náuseas y vómitos (n = 100). Paraná, Brasil, 2023

**Variables**	**Auriculoterapia (G1)**	**Láser (G2)**	**Control (G3)**	**Placebo (G4)**	**Valor p**
PUQE* 1 ^‡^	8,4 (7,8; 9,1)	8,2 (7,4; 8,9)	8,7 (8,0; 9,3)	8,4 (7,8; 9,1)	0,782
PUQE* 2 ^‡^	7,8 (7,1; 8,5)	7,5 (6,7; 8,2)	8,7 (8,1; 9,4)	8,5 (7,8; 9,2)	0,042
PUQE* 3 ^‡^	6,9 (6,3; 7,5)	6,8 (6,1; 7,5)	8,5 (7,9; 9,1)	8,4 (7,8, 9,1)	<0,001
PUQE* 4 ^‡^	5,9 (5,3; 6,5)	5,8 (5,1; 6,5)	8,5 (7,9; 9,1)	8,6 (7,9; 9,2)	<0,001
NVPQOL ^†^ 1 ^‡^	121,5	127,8	134,6	137,0	0,075
	(114,0; 128,9)	(117,5; 138,2)	(126,4; 142,8)	(127,9; 146,2)	
NVPQOL ^†^ 2 ^‡^	86,0	85,8	133,4	139,6	<0,001
	(79,4; 92,7)	(77,8; 93,8)	(124,5; 142,4)	(131,1; 148,1)	
Palpación 1 ^§^					0,387
Grado I	0 (-)	0 (-)	0 (-)	1 (100,0)	
Grado II	25 (25,3)	25 (25,3)	25 (25,3)	24 (24,1)	
Palpación 2 ^§^					<0,001
Grado I	14 (43,8)	16 (50,0)	1 (3,1)	1 (3,1)	
Grado II	11 (16,2)	9 (13,2)	24 (35,3)	24 (35,3)	
Dosisde metoclopramida utilizada (mg)*	2,8	5,2	40,0	42,4	<0,001
	(0,6; 5,0)	(1,6; 8,8)	(28,2; 51,8)	(30,2; 54,6)	
Uso continuo de sulfato ferroso ^§^					0,243
Sí	20 (24,1)	20 (24,1)	19 (22,9)	24 (28,9)	
No	5 (29,4)	5 (29,4)	6 (35,3)	1 (5,9)	

*PUQE = *Pregnancy Unique Quantification of Emesis*; ^†^NVPQOL = *Health-Related Quality of Life Questionnaire for Nausea and Vomiting of Pregnancy*; ^‡^Media (intervalo de confianza del 95%); ^§^n (%)

Se observó que las mujeres embarazadas de los grupos G3 y G4 hicieron, estadísticamente, mayor uso de metoclopramida en comparación con los grupos de intervención (Tabla 2). También se observó que la mayoría de las participantes utilizaba de forma continua sulfato ferroso en la dosis de 40 mg/día para la prevención de anemia gestacional, sin diferencias estadísticas entre los grupos.

En el análisis del puntaje general del cuestionario NVPQOL antes de las intervenciones, se registraron puntuaciones más altas en los ítems 1, 4, 8, 15 y 29, que corresponden, respectivamente, a náuseas, ganas de vomitar, sentirse peor con ciertos olores, menor interés en las relaciones sexuales y dificultad para cocinar. Estos hallazgos señalan que los síntomas de las NVE pueden afectar diversos ámbitos de la vida de la mujer embarazada y de su familia, y que el olfato puede influir en el agravamiento de los síntomas. Por otro lado, los ítems que presentaron puntuaciones más bajas antes de la intervención (6, 19, 20 y 21), que corresponden, respectivamente, al empeoramiento de los síntomas en la tarde, la percepción de que los síntomas no son parte de un embarazo normal, la sensación de no poder disfrutar del embarazo y el hecho de que todo supone un esfuerzo, indican que, a pesar de los síntomas desagradables, gran parte de las mujeres embarazadas afronta las NVE como un evento fisiológico del embarazo.

Con respecto a la reducción en los puntajes después de la aplicación de la auriculoterapia y de la laserpuntura auricular, los ítems que mostraron mayor disminución fueron el 1, 4, 5, 8 y 9, que corresponden, respectivamente, a náuseas, ganas de vomitar, pérdida de apetito y sentirse peor con ciertos olores o alimentos. La reducción de estos valores indica que dichas terapias fueron eficaces para disminuir estos síntomas. Dos ítems mostraron escasa reducción con las intervenciones: el 6, que se refiere al empeoramiento de los síntomas en el período de la tarde —el cual ya había recibido baja puntuación antes de la intervención—, y el ítem 14, relacionado con sentirse emotiva, lo que puede explicarse por los cambios hormonales que ocurren al inicio del embarazo.

Las participantes de los grupos G1 y G2 presentaron, en modelos crudos, una reducción estadísticamente significativa en los puntajes de PUQE y NVPQOL en comparación con las de los grupos G3 y G4 (Tabla 3). Los resultados fueron, en su mayoría, replicados en el modelo ajustado por factores de confusión, con excepción del G2 en el NVPQOL, que perdió la significancia estadística al compararse con el G3.

En el modelo ajustado, al compararse con el G4, los grupos G1 y G2 redujeron (p < 0,050) los puntajes de PUQE en -1,14 (Intervalo de Confianza del 95%: -2,11; -0,17) y -1,3 (IC95%: -2,24; -0,41), y los puntajes de NVPQOL en -34,81 (IC95%: -62,98; -0,95) y -31,97 (IC95%: -62,98; -0,95), respectivamente (Tabla 3).

**Tabla 3-:** Evaluación de la eficacia de la auriculoterapia y la laserpuntura auricular en relación con las puntuaciones PUQE* y NVPQOL^†^, entre las embarazadas con náuseas y vómitos (n = 100). Paraná, Brasil, 2023

**Modelo bruto**	**Control (G3)**	**En comparación con el grupo de control**		
		**Placebo** ^‡^ **(G4)**	**Auriculoterapia** ^‡^ **(G1)**	**Laserpuntura** ^‡^ **(G2)**
PUQE*	0,00	-0,13 (-0,25; -0,01. 0,027)	-1,34 (-2,19; -0,49. 0,002)	-1,55 (-2,36; -0,74. <0,001)
NVPQOL ^†^	0,00	4,26 (1,68; 6,84. 0,001)	-30,28 (-51,01; -6,55. 0,012)	-27,22 (-55,52; 1,08. 0,059)
**Modelo ajustado** ^§^	**Control (G3)**	**Placebo** ^‡^ **(G4)**	**Auriculoterapia** ^‡^ **(G1)**	**Laserpuntura** ^‡^ **(G2)**
PUQE*	0,00	-0,26 (-0,39; -0,13. <0,001)	-1,40 (-2,25; -0,54. 0,001)	-1,59 (-2,38; -0,79. <0,001)
NVPQOL ^†^	0,00	5,99 (3,26;8,73)	-28,82 (-52,64; -4,99. 0,018)	-25,97 (-54,25; 2,31. 0,072)
**Modelo bruto**	**Placebo (G4)**	**En comparación con el grupo placebo**		
		**Control** ^‡^ **(G3)**	**Auriculoterapia** ^‡^ **(G1)**	**Laserpuntura** ^‡^ **(G2)**
PUQE*	0,00	0,13 (0,01; 0,25. 0,027)	-1,21 (-2,18; -0,24. 0,014)	-1,42 (-2,34; -0,50. 0,002)
NVPQOL ^†^	0,00	-4,26 (-6,84; -1,68)	-34,54 (-60,84; -8,24. 0,010)	-31,48 (-62,36; -0,60. 0,046)
**Modelo ajustado** ^§^	**Placebo (G4)**	**Control** ^‡^ **(G3)**	**Auriculoterapia** ^‡^ **(G1)**	**Laserpuntura** ^‡^ **(G2)**
PUQE*	0,00	0,26 (0,13; 0,39. <0,001)	-1,14 (-2,11; -0,17. 0,022)	-1,32 (-2,24; -0,41. 0,005)
NVPQOL ^†^	0,00	-5,99 (-8,73; -3,26. <0,001)	-34,81 (-62,98; -0,95. 0,043)	-31,97 (-62,98; -0,95. 0,043)

*PUQE = *Pregnancy Unique Quantification of Emesis*; ^†^NVPQOL = *Health-Related Quality of Life Questionnaire for Nausea and Vomiting of Pregnancy*; ^‡^Beta (intervalo de confianza del 95%. Valor p); ^§^Modelo ajustado por edad, edad gestacional, índice de masa corporal, paridad y número de consultas

## Discusión

La auriculoterapia y la laserpuntura auricular fueron eficaces en la reducción de las NVE y en la mejora de la calidad de vida de las mujeres embarazadas. No obstante, en el modelo ajustado, el grupo G2 en comparación con el G3 no presentó una mejora estadísticamente significativa en la calidad de vida, aunque sí se observó una reducción en los puntajes del NVPQOL, lo que nos lleva a sugerir la realización de nuevas investigaciones con una población mayor.

La calidad de vida de las mujeres embarazadas puede verse afectada por la relación con la pareja y la familia, ya que los conflictos conyugales pueden considerarse factores etiológicos de ansiedad y depresión en este período, emociones capaces de intensificar los síntomas de las NVE^(29)^. El bienestar de la mujer embarazada también puede estar relacionado con la vida sexual activa durante este tiempo; sin embargo, síntomas como las NV pueden influir negativamente en la libido^(30)^, lo que en el presente estudio se reflejó en el menor interés en las relaciones sexuales.

Otros factores pueden intensificar la presencia de NVE, como las cuestiones hormonales, una alimentación con alto contenido de grasas o condimentos, y ciertos olores, dado que el olfato es más sensible en las mujeres durante el primer trimestre. En este sentido, una dieta saludable se asocia con menor riesgo de desarrollar hiperémesis gravídica^(31)^.

El olfato más agudo en mujeres en el primer trimestre del embarazo está relacionado con el procesamiento cognitivo de la información olfativa y se considera más alterado que el gusto. Así, el olor puede tener un mayor potencial para provocar los síntomas de las NVE^(31)^, lo que confirma los hallazgos del presente estudio, ya que las mujeres informaron que percibir ciertos olores provoca más NV que el hecho de comer ciertos alimentos.

Uno de los ítems que tuvo menor puntuación antes de la intervención y prácticamente no presentó cambios después de las terapias fue el empeoramiento de los síntomas de las NVE en el período de la tarde. Esto se debe a que los síntomas suelen intensificarse en la mañana, como resultado del ayuno prolongado durante la noche, dado que la producción de jugo gástrico aumenta y puede refluir hacia el esófago, ocasionando las NVE^(2)^.

En este estudio se observaron puntuaciones más bajas en los ítems relacionados con la percepción de que los síntomas de las NV no corresponden a un embarazo normal, con la sensación de no poder disfrutar de la gestación y con la idea de que todo resulta un esfuerzo cuando se relaciona con la presencia de NVE. Estos resultados permiten afirmar que las mujeres mostraron resiliencia frente al malestar causado por las NVE, percibiendo esta situación como una alteración fisiológica propia del embarazo.

A pesar de que las participantes demostraron resiliencia frente a las molestias físicas, informaron dificultades para mantener el trabajo, las actividades diarias y las actividades sociales. Sin embargo, tras la aplicación de las terapias, se observó una disminución de estos reportes, lo que demuestra que el tratamiento precoz contribuye a la mejora de la calidad de vida y reduce las probabilidades de evolución hacia un cuadro de hiperémesis gravídica. El tratamiento precoz es particularmente importante si se considera que la hiperémesis gravídica, con gran repercusión en las actividades diarias y sociales, genera sufrimiento, incapacidad funcional e influye en la decisión sobre futuros embarazos^(32)^.

El sulfato ferroso también puede influir en la aparición de las NVE, ya que este es un efecto secundario de la medicación^(33)^. No obstante, dado que este suplemento es prescrito a todas las mujeres embarazadas conforme a la orientación del Ministerio de Salud, con el fin de prevenir la anemia ferropénica, se considera que su uso no interfirió en los resultados encontrados. De hecho, no hubo diferencias en el patrón de uso entre las mujeres de los cuatro grupos.

Independientemente de los factores asociados a las NVE y con el objetivo de aliviar los síntomas de esta afección, es posible utilizar métodos farmacológicos y no farmacológicos. Como método farmacológico, el uso de antieméticos, como la metoclopramida, fue una variable de interés en el presente estudio. En Noruega, una cohorte retrospectiva observó las implicaciones del uso de antieméticos durante el embarazo, las cuales se asociaron con efectos secundarios fetales, como un mayor riesgo de fisuras orofaciales y la tendencia a hospitalización temprana en la gestación^(3)^.

También en Noruega, un estudio reportó la asociación entre los síntomas de NVE y la multiparidad, lo que no pudo ser observado en el presente estudio, dado que todos los grupos estuvieron constituidos, en su mayoría, por multíparas^(34)^. Sin embargo, los hallazgos de esta investigación corroboran los de un estudio nacional en el que se constató que las terapias complementarias disminuyen el consumo y los daños provocados por las medicaciones alopáticas^(35)^.

La auriculoterapia es un método no farmacológico relevante, pues además de ser práctico y de bajo costo, ha demostrado reducir los síntomas de las NVE sin necesidad de medicación^(36)^, sobre todo cuando se utiliza al inicio de los síntomas. Este beneficio también pudo observarse en el presente estudio, dado que gran parte de las mujeres embarazadas iniciaron el control prenatal en una edad gestacional adecuada, alrededor de la octava semana. Otros métodos no farmacológicos descritos en la literatura y que pueden utilizarse en los casos leves son la hidroginástica, el uso de vitamina B6 y la ingesta de jengibre^(2,37-38)^. Estas terapias fortalecen la práctica de enfermería en las UBS, pues promueven la integralidad del cuidado y el rescate de los saberes populares. Se trata de un escenario en el que los profesionales se ven impulsados a incorporar en su rutina laboral la educación en salud, con el fin de implementar prácticas menos invasivas y medicalizadas^(35)^, especialmente en el contexto del control prenatal.

La educación en salud sobre estas terapias promueve el autoconocimiento de las mujeres y la construcción del concepto de salud, otorgándoles un papel activo en el proceso salud-enfermedad y fomentando la reflexión sobre el acceso a dichas terapias en el ámbito del sistema público de salud^(35)^. De este modo, las terapias complementarias son consideradas tecnologías blandas en la innovación del cuidado y en el tratamiento de las NVE, y resultan relevantes para el restablecimiento de la calidad de vida de las mujeres embarazadas. Por lo tanto, se sugiere la incorporación de estas terapias en el cuidado de enfermería, dado que son prácticas menos invasivas y mejor aceptadas por sus efectos positivos^(8)^.

El uso de la auriculoterapia en casos leves ya está recomendado por la FEBRASGO; sin embargo, en este estudio, los valores promedio del puntaje PUQE antes de la intervención indicaban un grado moderado de NVE, y los dos grupos de intervención mostraron una reducción significativa de dicho puntaje después de 96 horas de intervención. Esto puede explicarse porque la auriculoterapia requiere un tiempo mínimo de entre 3 y 4 días para alcanzar el pico máximo de su acción, reduciendo su efectividad después de ese período, hasta el séptimo día^(37)^. No se encontraron registros en la literatura sobre el tiempo de eficacia de la laserpuntura auricular; sin embargo, en el presente estudio, dicho tiempo fue similar al de la auriculoterapia.

Tampoco se encontraron registros en la literatura sobre el efecto de la laserpuntura auricular en las NVE. No obstante, los hallazgos de este estudio demuestran que su uso es tan eficaz como el de las agujas semipermanentes para el tratamiento de estos síntomas, tanto en casos leves como moderados. Esto podría favorecer su elección entre los métodos no farmacológicos, dado que, al ser indolora, posee un gran potencial de aceptación entre las mujeres embarazadas. La eficacia similar se debe probablemente al efecto antiinflamatorio de la estimulación del nervio vago en el pabellón auricular^(39)^.

Los estímulos auriculares con agujas semipermanentes son capaces de reducir los niveles séricos de proteína C-reactiva (PCR), factor de necrosis tumoral-α (TNFα), interleucina (IL)-6 e IL-10^(6,39-40)^, involucrados en el proceso inflamatorio y en el dolor. La vía de señalización responsable de esta reducción se regula mediante la estimulación periférica del nervio vago, que promueve la respuesta antiinflamatoria^(39)^. La fotobiomodulación inducida por la utilización del láser de baja potencia, en la misma longitud de onda utilizada en esta propuesta, ha demostrado eficacia en la señalización antiinflamatoria con la consiguiente reducción de TNF-α e interleucinas IL-1β, IL-8 e IL-12^(41)^.

A pesar de los resultados prometedores del estudio, deben considerarse algunas limitaciones, tales como: la imposibilidad de cegar a las participantes y a la acupunturista; la falta de análisis de subgrupos; el hecho de no haber evaluado el tiempo de respuesta de la laserpuntura auricular; el grado de estrés y ansiedad de las mujeres embarazadas en los dos momentos de la evaluación; los efectos adversos de manera sistemática; y el tiempo reducido de seguimiento para verificar la permanencia de los beneficios obtenidos.

Se recomienda que futuros estudios tengan un alcance mayor (multicéntricos) e incluyan la evaluación de los costos en la aplicación de las técnicas de auriculoterapia y laserpuntura auricular, la aceptabilidad de las participantes, los hábitos alimentarios y un diseño metodológico que permita evaluar la eficacia de la intervención a largo plaz.

## Conclusión

Los resultados del estudio demostraron que la auriculoterapia y la laserpuntura auricular son métodos no farmacológicos eficaces para la reducción de las NVE y la mejora de la calidad de vida. Asimismo, que la laserpuntura auricular es tan eficaz como la auriculoterapia en la reducción de las NVE y que, al no generar molestias en su aplicación, puede tener una mayor aceptabilidad entre las mujeres embarazadas, contribuyendo así a la incorporación de las prácticas complementarias como métodos no farmacológicos para disminuir estos síntomas.

El uso de estas terapias también contribuyó a la reducción del consumo de medicamentos alopáticos por parte de las mujeres embarazadas y a la mejora de la calidad de vida, lo que implica la posibilidad de restablecimiento de las actividades laborales, cotidianas y sociales.

## Data Availability

Todos los datos generados o analizados durante este estudio están incluidos en este artículo publicado.
